# Immune and oxidative stress biomarkers in pediatric psychosis and psychosis-risk: Meta-analyses and systematic review

**DOI:** 10.1016/j.bbi.2023.12.019

**Published:** 2023-12-21

**Authors:** Jerome Henry Taylor, Julieta Bermudez-Gomez, Marina Zhou, Oscar Gómez, Casey Ganz-Leary, Cesar Palacios-Ordonez, Zeeshan M. Huque, Ran Barzilay, David R. Goldsmith, Raquel E. Gur

**Affiliations:** a Children’s Hospital of Philadelphia (CHOP), Philadelphia, PA, USA; b University of Pennsylvania Perelman School of Medicine, Philadelphia, PA, USA; c Lifespan Brain Institute of CHOP and Penn Medicine, Philadelphia, PA, USA; d National Institute of Psychiatry Ramon de la Fuente Muñiz, Mexico City, Mexico; e Statiscripts, LLC, USA; f Faculty of Medicine, Pontificia Universidad Javeriana, Bogotá, Colombia; g Temple University, Philadelphia, PA, USA; h Emory University School of Medicine, Atlanta, GA, USA; i Monterrey Institute of Technology and Higher Education, Monterrey, Mexico

**Keywords:** Early-onset psychosis, Schizophrenia, Children and adolescents, Inflammation, Reduction–oxidation

## Abstract

**Objective:**

While genetic and cohort studies suggest immune and reduction/oxidation (redox) alterations occur in psychosis, less is known about potential alterations in children and adolescents.

**Methods:**

We conducted a systematic review to identify immune and redox biomarker studies in children and adolescents (mean age ≤ 18 years old) across the psychosis spectrum: from psychotic like experiences, which are common in children, to threshold psychotic disorders like schizophrenia. We conducted *meta*-analyses when at least three studies measured the same biomarker.

**Results:**

The systematic review includes 38 pediatric psychosis studies. The *meta*-analyses found that youth with threshold psychotic disorders had higher neutrophil/lymphocyte ratio (Hedge’s *g* = 0.40, 95 % CI 0.17 – 0.64) tumor necrosis factor (Hedge’s *g* = 0.38, 95 % CI 0.06 – 0.69), C-reactive protein (Hedge’s *g* = 0.38, 95 % CI 0.05 – 0.70), interleukin-6 (Hedge’s *g* = 0.35; 95 % CI 0.11 – 0.64), and total white blood cell count (Hedge’s *g* = 0.29 95 % CI 0.12 – 0.46) compared to youth without psychosis. Other immune and oxidative stress *meta*-analytic findings were very heterogeneous.

**Conclusion:**

Results from several studies are consistent with the hypothesis that signals often classified as “proinflammatory” are elevated in threshold pediatric psychotic disorders. Data are less clear for immune markers in subthreshold psychosis and redox markers across the subthreshold and threshold psychosis spectrum. Immune and redox biomarker intervention studies are lacking, and research investigating interventions targeting the immune system in threshold pediatric psychosis is especially warranted.

## 1. Introduction

Many children and adolescents with early-onset (before age 18) schizophrenia or schizoaffective disorder do not respond to gold-standard antipsychotic medications (Findling et al., 2010; Frazier et al., 2012; Gabriel et al., 2017; Sikich et al., 2008; Taylor et al., 2021). Even when antipsychotics reduce psychosis symptoms, residual symptoms often persist and there are frequently side effects, including extrapyramidal symptoms and weight gain (Findling et al., 2010; Sikich et al., 2008; Taylor et al., 2018). Clarifying the biological mechanisms related to psychosis-risk and psychosis is critical for the development of novel interventions for youth with and at risk for developing chronic and debilitating psychotic disorders.

“Subthreshold” psychosis symptoms commonly precede the diagnosis of the more severe “threshold” psychotic disorders (TPDs), defined in Table 1. Subthreshold psychosis includes psychotic like experiences (PLEs) (Barzilay et al., 2018; Dong et al., 2021; Taylor et al., 2020b), which can be developmentally normative in childhood. Subthreshold psychosis also includes attenuated psychosis symptoms when they are part of “at risk mental states” for transdiagnostic psychiatric outcomes (McGorry et al., 2018) and psychosis-risk syndromes, like clinical high risk for psychosis (CHR–P, sometimes referred to as ultra-high risk for psychosis) (Catalan et al., 2021; Miller et al., 1999; Taylor et al., 2020b; Taylor and Huque, 2021; Woods et al., 2018; Yung et al., 2005). Individuals who have abiological family member with TPD are considered at familial high-risk for psychosis (FHR-P); individuals with a first-degree relative with TPD have a five- to ten-fold elevated risk of developing a TPD (Rasic et al., 2014; Taylor et al., 2020a). TPDs are most commonly diagnosed between ages 20 and 34 years old (Solmi et al., 2022) and are characterized by hallucinations, delusions, and impaired functioning. Schizophrenia spectrum disorders (SCZs), such as schizophrenia and schizoaffective disorder, are prototypical TPDs (Gur, 2022).

While there is evidence for several genetic and environmental factors that contribute to psychosis, the precise pathophysiology of psychosis remains unknown (Coury et al., 2023; Davies et al., 2020; Galdos et al., 1993; Meltzer and Stahl, 1976; Muhrer et al., 2022; Perkins et al., 2015). Recent *meta*-analyses in adults confirm elevations in peripheral immune markers in acute and chronic schizophrenia compared to healthy controls (Halstead et al., 2023) and oxidative stress markers in adults with threshold psychosis compared to healthy controls (Jorgensen et al., 2022). However, the evidence focused on the pediatric population, that is children and adolescents under age 18, is sparse. In fact, a most recent *meta*-analysis and systematic review focused on pediatric psychosis only identified seven studies for inclusion and did not find differences in immune or oxidative stress markers when comparing pediatric patients with threshold psychosis to healthy controls (Fraguas et al., 2017). A focus on studies of individuals under age 18 is warranted given that assessment and treatment guidelines for psychosis often vary based on the age 18 cutoff (McClellan and Stock, 2013; National Institute for Health and Care Excellence, 2016). Also, while adolescent and adult immune systems are very similar, there are some differences, including adolescents tending to have higher percentages of white blood cells (WBCs) that are lymphocytes and monocytes (Valiathan et al., 2016). Similarly, prior work has found that higher age is associated with significantly lower antioxidant status, even within adolescence (Mico et al., 2011). It is important to note that while some markers are typically associated with “proinflammatory” states, many immune cells and signals can have pro- and anti-inflammatory effects, depending on the context. Similarly, while some redox markers may be classically associated with “oxidative stress,” balance between oxidation and reduction processes is key.

The current systematic review and *meta*-analysis focuses on pediatric studies (mean age ≤ 18 years old). It includes studies through June 1, 2023, updating the findings from a most recent pediatric-focused systematic review and *meta*-analysis on inflammation and oxidative stress in threshold psychosis that was conducted through October 2016 (Fraguas et al., 2017). Moreover, the current work reviews subthreshold psychosis and psychosis-risk studies in addition to threshold psychosis studies. We sought to review the evidence for two hypotheses: 1) Immune and oxidative stress biomarkers would be altered in TPD in the pediatric population, and 2) Immune and oxidative stress biomarkers altered in TPD would also be altered in subthreshold psychosis and psychosis-risk states (FHR-P, PLEs, and CHR-P), even if to a lesser extent.

## 2. Methods

### 2.1. Search strategy

We conducted a systematic search in MEDLINE/PubMed and Web of Science from inception through June 1, 2023. We followed the recommendations by the Preferred Reporting Items for Systematic Reviews and Meta-Analysis (PRISMA) guidelines (Page et al., 2021), and the search strategy for each database is available in the online supplementary materials. The study was pre-registered in PROSPERO (https://www.crd.york.ac.uk/prospero) with identification number CRD42022296517.

### 2.2. Study screening and selection

A total of 2,631 citations were retrieved and then imported into and collated in the free software application Rayyan for systematic reviews (Ouzzani et al., 2016). Three reviewers (JB, CP, and OG) independently screened records to assess eligibility, and disagreements were resolved by consensus among reviewers. If a consensus could not be reached, an additional investigator (JHT) made the final decision on inclusion. We included studies that: a) were conducted in children and adolescents (mean age ≤ 18 years old) along the psychosis and psychosis-risk continuum (including FHR-P, PLE, CHR-P, and TPD); b) measured an immune and/or oxidative stress biomarker; c) were published either in English or Spanish. Notably, we did not require a comparison group without psychosis/psychosis-risk for inclusion in the systematic review; however, we did require a comparison group without psychosis/psychosis-risk for inclusion in *meta*-analyses. We excluded studies published before 1971, when the human enzyme-linked immunosorbent assay was discovered, consistent with prior *meta*-analyses (Walsh et al., 2023).

### 2.3. Data extraction

Data was extracted by four investigators (OG, JB, CG, and MZ) in an Excel spreadsheet and reviewed by a second investigator (OG or JB). For each study, we obtained the following information: year of publication, study design (case-control, cohort, or randomized-controlled trial), sample size, age, gender, race/ethnicity, categorization of psychosis continuum group, type of biomarker, type of biospecimen [blood (cells, plasma, serum), cerebrospinal fluid (CSF), urine, or salivary], quantitative measures of biomarkers, and potential moderators – body mass index (BMI), smoking status as percentage, use of antipsychotic medication, and duration of psychosis symptoms.

### 2.4. Quality assessment

Quality was assessed independently by two investigators (OG and CG or OG and JB) using the Newcastle-Ottawa Scale for case-control and cohort studies (Wells et al., 2013). We awarded one point in item 1 of the exposure/outcome section if a study provided enough details of the biomarker measurement method (i.e. laboratory technique, method of collection and storage, and reported coefficient of variation). There is no consensus on the interpretation of Newcastle-Ottawa Scale scores; for the purposes of this paper, we classified a study as being of high to moderate quality if the total score was ≥ 7 and at least 1 point was awarded for each of the three sections. For randomized-controlled trials, the RoB 2.0 tool was used to assess quality (Sterne et al., 2019). Disagreements in ratings were resolved through a consensus discussion.

### 2.5. Statistical analysis

Statistical analysis was performed in STATA 16.0 software. Meta-analyses were based on the means and standard deviations of redox and immune biomarker levels. We calculated Hedge’s g effect sizes due to the likelihood of small clinical samples. When needed, we calculated standard deviations from standard errors using $SD=SE\times \sqrt{N}$ (Higgins et al., 2022), and in the case of medians and interquartile ranges, we followed earlier published recommendations (Greco et al., 2015; Wan et al., 2014). We conducted random-effects *meta*-analyses for biomarkers with quantitative data in at least three studies. A random-effects analysis was preferred over fixed-effects due to expected high heterogeneity between studies, as observed in a similar *meta*-analysis (Fraguas et al., 2019). In longitudinal cohorts that investigated biomarker associations with psychosis symptoms at multiple time points, we included the time point at which the youth was oldest in the *meta*-analysis because threshold psychotic disorders are more likely to present later in adolescence, and potentially developmentally-normative PLEs reported in childhood often subside later in adolescence (Calkins et al., 2017; Kelleher et al., 2012). To assess heterogeneity in effect estimates, the *I*^2^ statistic was used, and we considered values below 40 % as low heterogeneity (Higgins et al., 2022). To assess publication bias, we used funnel plots and the trim-and-fill method to adjust estimations in case of asymmetry. In addition, regression-based Egger test was used to detect small-study effects. When there was sufficient data, we conducted *meta*-regression analysis to examine potential moderating effects of age, identified sex, BMI, cigarette smoking status (as percentage), antipsychotic status, and study quality assessment score.

## 3. Results

**Fig. 1.**
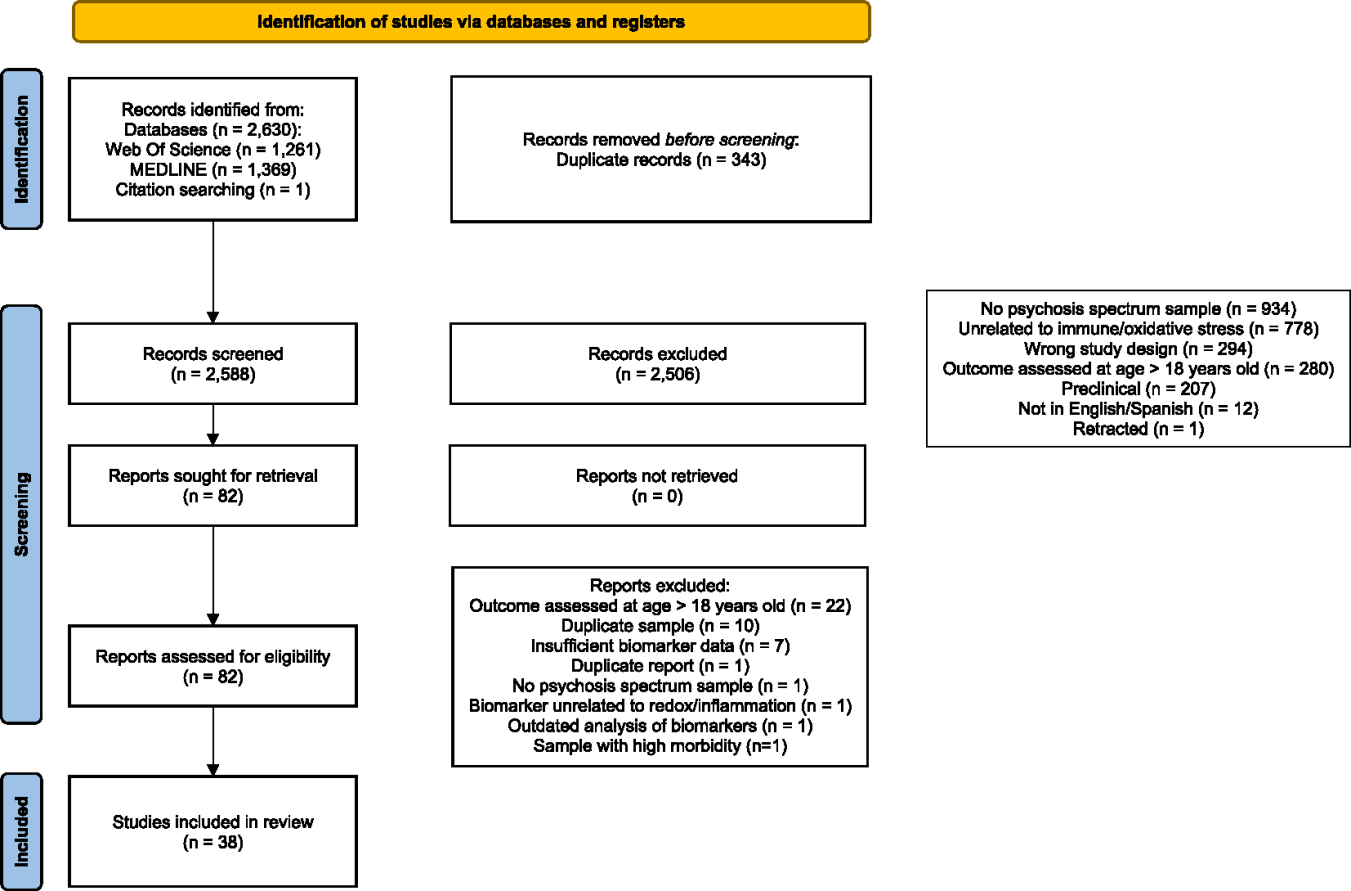
PRISMA flow diagram of the identification and selection of studies.

**Fig. 2.**
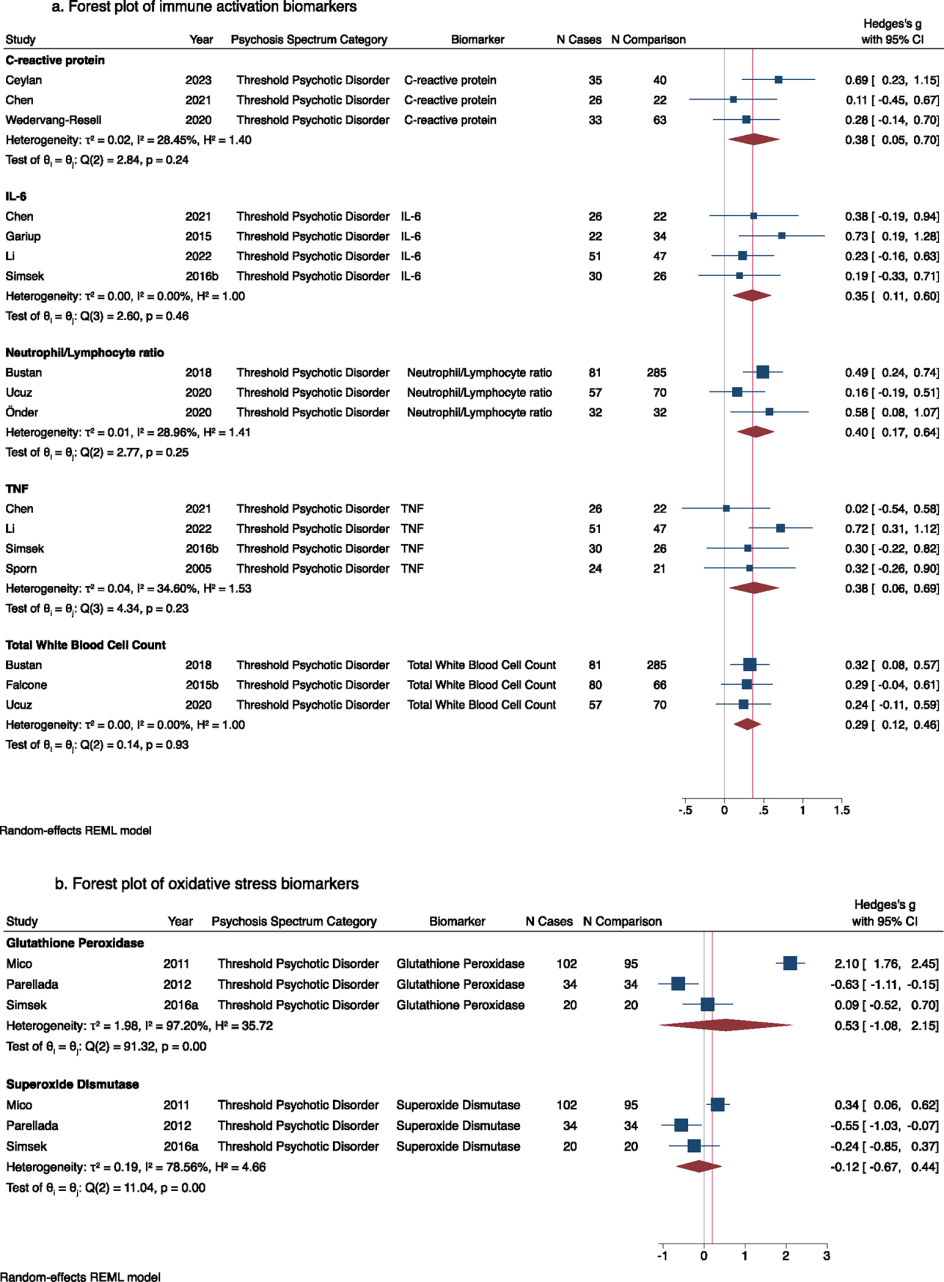
a) Forest plot of immune biomarkers in threshold psychosis, b) Forest plot of oxidative stress biomarkers in threshold psychosis.

The systematic review identified 38 pediatric psychosis studies meeting inclusion criteria (Fig. 1) (Amminger et al., 2010; Berger et al., 2016, 2020; Bustan et al., 2018; Ceylan et al., 2023; Chen et al., 2021; Cullen et al., 2017; English et al., 2018; Falcone et al., 2015b, 2015a; Focking et al., 2016, 2021; Garcia et al., 2018; Gariup et al., 2015; Gonzalez-Pinto et al., 2012; Khandaker et al., 2014a, 2021; Li et al., 2022; Lizano et al., 2016; Lundberg et al., 2022; Madrid-Gambin et al., 2019; Mico et al., 2011; Mittleman et al., 1997; Moreno et al., 2019; O’Gorman et al., 2017; Onder et al., 2020; Parellada et al., 2012; Simsek et al., 2016a, 2016b; Smesny et al., 2017; Sporn et al., 2005; Trotta et al., 2021; Ucuz et al., 2020; Wake et al., 2022; Wedervang-Resell et al., 2020; Xu et al., 2016; Zeni-Graiff et al., 2019; Zhang et al., 2022). Supplementary Table 1 describes each study from the systematic review, Supplementary Table 2 lists studies for each biomarker, Supplementary Table 3 describes biospecimen storage and collection procedures for the reviewed studies, and Supplementary Fig. 1 quantifies effect sizes for all studies with psychosis/psychosis-risk cases and non-psychosis/psychosis-risk comparisons categorized into immune, oxidative stress, bioactive lipids, and other biomarkers related to immune and redox pathways. We included the immune biomarkers basophils, C-reactive protein (CRP), cortisol, eosinophils, granulocyte–macrophage colony-stimulating factor (GM-CSF), homocysteine, interferon-γ (IFN-γ), IFN-γ-induced protein-10 (IFN-γ-IP-10), interleukin-1β (IL-1β), IL-2, IL-4, IL-5, IL-6, IL-8, IL-10, IL-17a, IL-18, IL-18/IL-18 binding protein (IL-18BP) ratio, IL-18BP isoform a (IL-18BPa), IL-18 receptor 1 (IL-18R1), IL-18 receptor accessory protein (IL-18RAP), lymphocytes, lymphotoxin alpha (formerly known as TNF-β), monocyte chemoattractant protein 1 (MCP-1), monocytes, monocyte/lymphocyte ratio, mean platelet volume (MPV), neutrophils, neutrophil/lymphocyte ratio, p40 subunit of IL-12 and IL-23 (IL-12-IL-23-p40), platelets, platelet/lymphocyte ratio, tumor necrosis factor (TNF, also known as TNF-α), soluble form of the intercellular adhesion molecule 1 (sICAM-1), soluble urokinase plasminogen activator receptor (suPAR), and total white blood cell count (WBC). We included the oxidative stress biomarkers biopyrrin/free immunoglobulin light chains (FLC) ratio, biopyrrin/creatinine (Cre) ratio, 8-hydroxy-2′-deoxyguanosine (8OhdG)/Cre ratio, coenzyme Q, catalase, glutathione, glutathione peroxidase, inducible nitric oxide synthase (iNOS), lipid hydroperoxides, superoxide dismutase, and total antioxidant status (TAS). We included the bioactive lipid biomarkers omega-3 and omega-6 polyunsaturated fatty acids, phosphatidylcholines, and lysophosphatidylcholines related to immunity and oxidative stress. Other biomarkers included peripheral S100B, related to blood brain barrier disruption, and brain derived neurotrophic factor (BDNF). In the following sections, we highlight findings for the seven biomarkers included in the *meta*-analyses (Fig. 2), other biomarkers implicated in psychosis in prior *meta*-analyses, and intervention studies related to biomarkers.

### 3.1. Immune activation

#### 3.1.1. Neutrophil/lymphocyte ratio

Youth with TPD had higher neutrophil/lymphocyte ratio (Hedge’s *g* = 0.40, 95%*CI* 0.17 – 0.64, *k* = 3, *N* = 557, *I*^2^ = 29.0%) compared to youth without psychosis in the *meta*-analysis (Bustan et al., 2018; Onder et al., 2020; Ucuz et al., 2020). Heterogeneity was low (*I*^2^ = 29.0%) in the neutrophil/lymphocyte ratio *meta*-analysis, even though the non-psychosis comparison groups were defined very differently in the studies: healthy adolescents presenting for a sports license (Onder et al., 2020), adolescents without psychiatric disorders determined by a child psychiatrist assessment (Ucuz et al., 2020), and adolescents on a psychiatric inpatient unit without psychotic or affective disorders (Bustan et al., 2018). No CHR-P, PLE, or FHR-P studies investigated neutrophil/lymphocyte ratio.

#### 3.1.2. WBC

Youth with TPD had higher WBC (Hedge’s *g* = 0.29, 95%*CI*0.12 – 0.46,*k* = 3,*N* = 639,*I*^2^ = 0%) compared to youth without psychosis in the *meta*-analysis (Bustan et al., 2018; Falcone et al., 2015b; Ucuz et al., 2020). There was no heterogeneity (*I*^2^ = 0%) for the WBC *meta*-analysis, even though the non-psychosis comparison groups were comprised of adolescents without psychiatric disorders (Ucuz et al., 2020), adolescents on inpatient psychiatric units without psychotic disorders (Falcone et al., 2015b), and adolescents on inpatient psychiatric units without psychotic or affective disorders (Bustan et al., 2018). No CHR-P, PLE, or FHR-P studies investigated total WBCs.

#### 3.1.3. TNF

Youth with TPD had higher TNF (Hedge’s *g* = 0.38, 95%*CI*0.06 – 0.69, *k* = 4, *N* = 247, *I*^2^ = 34.6%) compared to youth without psychosis in the *meta*-analysis (Chen et al., 2021; Li et al., 2022; Simsek et al., 2016b; Sporn et al., 2005). In the sole cerebrospinal fluid (CSF) study in the systematic review, CSF TNF was similar in youth with TPD (very early onset schizophrenia) compared to youth with obsessive compulsive disorder and attention deficit hyperactivity disorder (Mittleman et al., 1997). The data from Mittleman and colleagues (2017) was not included in *meta*-analyses because it was the only reviewed study to measure central nervous system biomarkers, as opposed to peripheral biomarkers. No studies compared TNF in youth with and without CHR-P, PLE, or FHR-P.

#### 3.1.4. IL-6

Youth with TPD had higher IL-6 (Hedge’s *g* = 0.35, 95%*CI*0.11 – 0.64,*k* = 4,*N* = 258,*I*^2^ = 0%) compared to youth without psychosis in the *meta*-analysis (Chen et al., 2021; Gariup et al., 2015; Li et al., 2022; Simsek et al., 2016b). However, the data in subthreshold psychosis studies was less clear. Specifically, the largest subthreshold psychosis study to investigate IL-6 is part of the Avon Longitudinal Study of Parents and Children (ALSPAC), which found serum IL-6 at age 9 was associated with PLEs at age 18 (Khandaker et al., 2014a) but not age 13 (Khandaker et al., 2014b) after adjusting for many sociodemographic factors. Furthermore, in the E-Risk Longitudinal Twin Study, plasma IL-6 was not associated with PLEs at age 18 (Trotta et al., 2021). No studies compared IL-6 in youth with and without CHR-P or FHR-P.

#### 3.1.5. CRP

Youth with TPD had higher CRP (Hedge’s *g* = 0.38, 95%*CI*0.05 – 0.70, *k* = 3, *N* = 219, *I*^2^ = 28%) compared to youth without psychosis (Ceylan et al., 2023; Chen et al., 2021; Wedervang-Resell et al., 2020). Youth with subthreshold psychosis had similar levels of CRP compared to youth without psychosis (Hedge’s *g* = 0.15, 95%*CI*(−0.04) −0.33, *k* = 3, *N* = 4054, *I*^2^ = 58%); however, the data were too heterogeneous for clear *meta*-analytic interpretation (Cullen et al., 2017; Khandaker et al., 2014a; Trotta et al., 2021) (Supplementary Figure 3). The high heterogeneity in PLE studies was likely related to the use of salivary CRP in one study (Cullen et al., 2017) instead of plasma/serum CRP, the use of different PLE scales (Khandaker et al., 2014a; Trotta et al., 2021), and differences in age of PLE assessment and CRP assessment. For instance, CRP and PLE were assessed simultaneously at age 13 in Cullen et al., 2017 and at age 18 in Trotta et al., 2021; and in Khandaker et al., 2014a CRP was obtained at age 9 and PLE assessed at age 18. No studies compared CRP in youth with and without CHR-P or FHR-P.

#### 3.1.6. Monocytes

Two studies investigating monocytes were included in the review. The studies found positive effect sizes for monocytes: in adolescents with TPD compared to adolescents with other psychopathology on an inpatient unit (Falcone et al., 2015b) and in adolescents with TPD, specifically SCZ, on an inpatient unit compared to healthy controls (Ucuz et al., 2020) (Supplementary Table 1, Supplementary Fig. 1a).

#### 3.1.7. IL-18

Two studies investigated pro-inflammatory cytokine IL-18, and both found that IL-18 was higher in adolescents with TPD compared to adolescents without psychiatric diagnoses (Wedervang-Resell et al., 2020; Xu et al., 2016) (Supplementary Table 1, Supplementary Fig. 1a).

#### 3.1.8. IL-1β

Three studies in the systematic review investigated the pro-inflammatory cytokine IL-1β but lacked comparison groups without psychosis/psychosis-risk with quantitative means for *meta*-analysis. IL-1β was elevated in TPD compared to controls in one study (Gariup et al., 2015). In the other studies, IL-1β did not predict progression from CHR-P to TPD (Focking et al., 2016) or progression from FHR-P to TPD (Lizano et al., 2016) (Supplementary Table 1).

### 3.2. Oxidative stress

#### 3.2.1. Glutathione peroxidase

Youth with TPD had similar glutathione peroxidase levels compared to youth without psychosis but heterogeneity was high (Hedge’s *g* = 0.53, 95%*CI*(−1.08) −2.15, *k* = 3, *N* = 305, *I*^2^ = 97%) (Mico et al., 2011; Parellada et al., 2012; Simsek et al., 2016a). Red blood cell (RBC) glutathione peroxidase was elevated in TPD compared to controls in the Child and Adolescent First Episode Psychosis Study in Spain (CAFEPS) cohort and the effect size was large (Mico et al., 2011); in CAFEPS, 41.18 % had been receiving an antipsychotic, but antipsychotic status did not affect results. However, other studies found no significant differences in serum (Simsek et al., 2016a) or RBC (Parellada et al., 2012) glutathione peroxidase in TPD compared to controls. Similarly, there were no significant differences between plasma glutathione peroxidase comparing adolescents with and without second-degree FHR-P (Gonzalez-Pinto et al., 2012). Glutathione peroxidase is a family of antioxidant enzymes that protects against oxidative damage, and cellular glutathione peroxidase is often considered a marker of elevated oxidative stress, perhaps related to a potential compensatory upregulation of glutathione peroxidase when oxidative stress is high (Lubos et al., 2011). When glutathione peroxidase reduces reactive oxygen species, the reduced form of glutathione (GSH) is the electron donor and cofactor in the reaction and becomes glutathione disulfide (GSSG) after the reaction. Methodological choice of RBC (Mico et al., 2011; Parellada et al., 2012) versus serum (Simsek et al., 2016a) and plasma (Gonzalez-Pinto et al., 2012) glutathione peroxidase likely contributed to the high *meta*-analysis heterogeneity and discrepancies in results, as reported in a prior *meta*-analysis (Fraguas et al., 2019). No studies compared glutathione peroxidase in youth with and without CHR-P.

#### 3.2.2. Superoxide dismutase

Youth with TPD had similar superoxide dismutase levels compared to youth without psychosis but heterogeneity was high (Hedge’s *g* = −0.12, 95%*CI*(−0.67) −0.44, *k* = 3, *N* = 305, *I*^2^ = 79%). Similar to glutathione peroxidase, methodological choice of RBC (Mico et al., 2011) versus serum (Simsek et al., 2016a) and plasma (Gonzalez-Pinto et al., 2012; Parellada et al., 2012) superoxide dismutase likely contributed to the high *meta*-analysis heterogeneity, as previously reported in a prior *meta*-analysis (Fraguas et al., 2019). Plasma superoxide dismutase levels were similar in the single study comparing adolescents with and without second-degree FHR-P (Gonzalez-Pinto et al., 2012). No studies compared superoxide dismutase levels in youth with and without CHR-P.

### 3.3. Intervention studies

Fish oil is the only intervention that has been investigated in pediatric psychosis and measured immune and/or oxidative stress biomarkers. Specifically, a randomized controlled trial (RCT) in Vienna, Austria of 81 adolescents with CHR-P found lower one-year transition rates to TPD when randomized to 12 weeks of supplemental fish oil (n = 41) (1.2 g omega-3 polyunsaturated fatty acids and 7.6 mg of mixed tocopherol (vitamin E) daily) instead of the placebo coconut oil (n = 40) (Amminger et al., 2010). In the placebo arm of the study, the p40 subunit of IL-12 and IL-23 (IL-12-IL-23-p40) was the only of 40 neuroinflammation biomarkers in the plasma to predict transition to psychosis (Focking et al., 2016). However, in the full randomized control trial, fish oil did not affect plasma IL-12-IL-23-p40 subunit levels, IL-6, or the soluble alpha (Tac) subunit of the IL-2 receptor (sIL-2r) but did increase the circulating soluble form of the intercellular adhesion molecule 1 (sICAM-1) (Focking et al., 2016; Smesny et al., 2017). The effects of fish oil supplementation preventing transition from CHR-P to TPD (Amminger et al., 2010) have not been replicated in the pediatric population and contrast with the NEURAPRO RCT of 304 young adults with CHR-P, which found fish oil and placebo (paraffin oil) had similar one-year transition rates to TPD when all participants received cognitive behavioral case management (McGorry et al., 2017). In sum, further research on fish oil’s potential protective effects against psychosis symptom progression is needed.

### 3.4. Publication bias and meta-regression

There was no evidence of publication bias on visualization of funnel plots (Supplementary Fig. 2) and Egger’s test for WBC (*p* = 0.72), neutrophil/lymphocyte ratio (*p* = 0.87), or CRP (*p* = 0.64). There was asymmetry in TNF, glutathione peroxidase, and superoxide dismutase funnel plots suggesting a possible bias towards publishing findings with smaller effect sizes — TNF Egger’s test (*p* = 0.06), glutathione peroxidase Egger’s test (*p* = 0.29), and superoxide dismutase Egger’s test (*p* = 0.23). There was asymmetry in the funnel plot for IL-6 with a trend in Egger’s test (*p* = 0.38) towards publishing studies with larger effect sizes. No biomarker had at least 10 studies, so findings regarding publication biases for individual biomarkers should not be over-interpreted (Higgins et al., 2022).

Similarly, robust *meta*-regression analyses typically require at least 10 studies, which none of our individual biomarkers met, so we grouped inflammation biomarkers and grouped oxidative stress biomarkers for *meta*-regression (Supplementary Table 5, Supplementary Figure 1). For inflammation biomarkers as a group, *meta*-regression showed no significant effects for mean age, the percentage of males, BMI, percentage of cigarette smokers, percentage taking antipsychotics, study quality assessment score, or psychosis spectrum category (TPD versus subthreshold psychosis). For oxidative stress biomarkers as a group, there was insufficient data to analyze the effects of cigarette smoking and BMI, and the remaining variables showed no significant effects. The lack of significant *meta*-regression findings should be interpreted with caution because BMI, cigarette smoking, and antipsychotic medication were inconsistently reported as described in Supplementary Table 1. Moreover, combining distinct biomarkers into inflammation groups and oxidative stress groups may obscure effects pertinent to specific individual biomarkers.

### 3.5. Quality of the included studies

Most of the included case-control/cohort studies (28/37) were classified as having high to moderate quality. The only randomized controlled trial was classified as low risk of bias according to the RoB 2 tool (Supplementary Table 4).

## 4. Discussion

Our systematic review and *meta*-analysis investigated the evidence for associations between pediatric psychosis/psychosis-risk and biomarkers of immunity and oxidative stress. As hypothesized, *meta*-analyses found elevations of biomarkers often considered “proinflammatory,” specifically neutrophil/lymphocyte ratio, TNF, CRP, IL-6, and total WBC, in youth with threshold psychosis compared to youth without psychosis. In contrast to our hypothesis, CRP was not elevated in in youth with PLEs compared to youth without PLEs, though the findings were very heterogeneous, possibly related to differences in PLE scales and CRP sample collection method. Also, in contrast to our hypothesis, glutathione peroxidase and superoxide dismutase levels were similar in youth with and without threshold psychosis, but the findings were very heterogeneous, likely related to biospecimen collection differences. No other immune biomarkers or oxidative stress biomarkers had sufficient studies to conduct a *meta*-analysis in pediatric threshold or subthreshold psychosis. Additionally, it is notable that our systematic review and *meta*-analysis focused on studies with mean age less than or equal to 18, and there is little study overlap with other recent *meta*-analyses focused on young adults (Misiak et al., 2021) and adults (Halstead et al., 2023).

### 4.1. Comparing our immune results to findings in adult psychosis

A most recent *meta*-analysis of peripheral cytokines in adults found that several pro-inflammatory cytokines, including IL-6, TNF, and CRP, were elevated in acute and chronic schizophrenia spectrum disorders compared to healthy controls (Halstead et al., 2023), congruent with our findings in threshold pediatric psychosis. Also consistent with our findings, recent *meta*-analyses in primarily adults found that individuals with first episode and chronic schizophrenia had higher neutrophil/lymphocyte ratios (Karageorgiou et al., 2019) and total WBCs (Jackson and Miller, 2020) compared to controls. In summary, even though our *meta*-analysis only included a few studies for each biomarker, our findings in pediatric threshold psychosis are consistent with larger *meta*-analyses in the adult literature.

In terms of subthreshold psychosis, only CRP was assessed in at least 3 studies (all were PLE studies), and the methodological differences in terms of biospecimen type (saliva vs. plasma/serum), PLE scale, and age of PLE assessment and CRP measurement likely contributed to the high heterogeneity of study findings. In the pediatric literature, there was a complete lack of subthreshold psychosis studies comparing immune biomarkers in youth with and without CHR-P and first-degree FHR-P. Even in the adult literature, *meta*-analyses comparing individuals with and without subthreshold psychosis are limited. However, a recent *meta*-analysis found: IL-6 was higher in young adults with CHR-P compared to healthy controls but not FHR-P compared to healthy controls; CRP and other cytokines were not elevated in CHR-P or FHR-P; and there were no significant differences between young adults with CHR-P who subsequently developed threshold psychosis and young adults with CHR-P who did not develop threshold psychosis (Misiak et al., 2021).

### 4.2. Comparing our oxidative stress results to findings in adult psychosis

Our oxidative stress *meta*-analytic findings were very heterogeneous in threshold psychosis studies, and the literature in subthreshold psychosis and FHR-P for the pediatric population is sparse. Still, the largest oxidative stress study reviewed suggested high oxidative stress (low total antioxidant status, low RBC glutathione, high RBC glutathione peroxidase, high lipid hydroperoxides) in adolescents at baseline with first episode threshold psychosis, with large effect sizes compared to controls, but the results require replication because the findings are from a single cohort in Spain – CAFEPS (Mico et al., 2011). Consistent with the high oxidative stress and psychosis link at baseline in CAFEPS, CAFEPS also found that lower baseline RBC glutathione (indicating higher oxidative stress) in threshold psychosis youth was associated with cortical gray matter loss at two-year follow-up; total antioxidant status was not investigated (Fraguas et al., 2012). However, in contrast to the baseline data, longitudinal analysis of CAFEPS data found evidence that lower oxidative stress was associated with more psychosis and mania symptoms and poorer outcomes among adolescents with threshold psychosis over two-year follow-up (Garcia et al., 2018). In summary, the redox biomarker literature in pediatric psychosis has grown some since a recent *meta*-analysis (Fraguas et al., 2017) but remains underdeveloped.

Regarding adult studies, a most recent *meta*-analysis on oxidative stress in primarily young adults found multiple peripheral blood markers of oxidative stress (low total antioxidant status, low docosahexaenoic acid, high homocysteine) were higher in threshold psychosis than healthy controls (Fraguas et al., 2019). Similarly, a recent *meta*-analysis in primarily adults found higher levels of urine and blood markers of oxidative stress in individuals with threshold psychotic disorders and individuals with other psychiatric disorders compared to healthy controls (Jorgensen et al., 2022).

### 4.3. Gaps in the pediatric literature

Gaps in the pediatric psychosis immune and oxidative stress biomarker literature include a lack of racial/ethnic diversity as often categorized in the United States (US Census Bureau, 2020) and a lack of standardized measurement of traumatic experiences, which are associated with PLEs in youth (Barzilay et al., 2018; Dong et al., 2021). Additionally, many promising immune and oxidative stress markers of psychosis identified in the adult literature, including elevated homocysteine, nucleic acid oxidative stress markers, IL-1β, soluble IL-2 receptor (sIL2-r), and monocytes, and lower total antioxidant status and omega-3 fatty acids were understudied or altogether lacking in pediatric threshold and subthreshold psychosis (Fraguas et al., 2019; Jorgensen et al., 2022; Upthegrove et al., 2014). Blood F2-isoprostanes and urinary F2-isoprostanes, which are often regarded as the gold-standard oxidative stress biomarkers because they are relatively chemically stable compared to most oxidative stress markers, have defined normal values, and are associated with lifestyle and disease factors associated with oxidative stress like smoking and atherosclerosis (Milne, 2017), also merit investigation in pediatric psychosis but are lacking. Moreover, plasma malondialdehyde-modified low density lipoprotein (MDA-LDL), an oxidized version of low density lipoprotein cholesterol, is an oxidative stress marker that predicted progression of psychosis symptoms from CHR-P to threshold psychosis over two years in the North American Prodromal Longitudinal Study (NAPLS) (Perkins et al., 2015), and MDA-LDL has not been investigated in pediatric psychosis and merits study.

Furthermore, while several studies have reported indicators of increased blood brain barrier disruption in multiple psychiatric disorders including schizophrenia in adult populations (Futtrup et al., 2020), robust and replicated studies are lacking in the pediatric population. Similarly, studies of CSF (Wang and Miller, 2018) and neuroimaging studies localizing immune and oxidative stress markers to the central nervous system and even specific regions of the brain parenchyma using 7 T proton magnetic resonance spectroscopy (MRS) of glutathione, glutamate and glutamine have been performed in adults with psychosis (Murray et al., 2024; Sydnor and Roalf, 2020). Meanwhile, only one pediatric CSF study was identified in the current systematic review, and it lacked a control group without psychopathology (Mittleman et al., 1997).

There is also greater need for longitudinal studies of biomarkers in pediatric subthreshold and threshold psychosis. Existing longitudinal PLE studies demonstrate the value in follow-up of childhood studies into later adolescence, when PLEs are less common and may be more indicative of psychotic psychopathology (Kelleher et al., 2012). For instance, in ALSPAC, age 9 IL-6 was not associated with age 13 PLEs (Khandaker et al., 2014b) but was associated with age 18 PLEs (Khandaker et al., 2014a). Large population-based cohort studies of biomarkers, like ALSPAC, have not investigated transition to threshold psychosis as defined by Diagnostic and Statistical Manual of Mental Disorders (DSM) criteria. Such studies would help clarify biomarkers uniquely associated with threshold psychosis versus PLEs.

Extant pediatric TPD longitudinal studies have occurred in the context of antipsychotic treatment, and a prior study found decreasing neutrophil/lymphocyte ratio was associated with psychosis symptom reduction over 6 months of treatment on an inpatient unit in Israel (Bustan et al., 2018). In contrast to the identified higher level of oxidative stress in adolescents with FEP compared to controls at baseline, decreases in oxidative stress among adolescents with FEP were associated with psychosis and mania symptom worsening over two years in CAFEPS (Garcia et al., 2018). Future investigation of changes in redox and immune status when treating TPD in other cohorts may further clarify the role of biomarkers in psychosis beyond cross-sectional analysis.

Anti-inflammatory and antioxidant intervention studies would also expand the current understanding of immune and redox biomarkers in pediatric populations but were also lacking. Amminger and colleagues (2010) found supplemental fish oil high in omega-3 fatty acids, which have anti-inflammatory and antioxidant properties, reduced one-year transition from CHR-P to threshold psychosis in adolescents, although the finding was not replicated in a larger sample of young adults (McGorry et al., 2017). Results have also been inconsistent when investigating non-steroidal anti-inflammatory drugs as adjunctive treatments in adult populations with schizophrenia, with some studies suggesting greater symptom improvement in first episode schizophrenia than chronic schizophrenia (Nitta et al., 2013; Zheng et al., 2017). There have been promising studies of supplements with antioxidant effects like ginko in adults with schizophrenia (Singh et al., 2010) and N-acetlycysteine in a subgroup of patients with high blood cell glutathione peroxidase (Conus et al., 2018), while other antioxidants like vitamin E and vitamin C (Firth et al., 2017) did not reduce symptoms in adults with schizophrenia. Future research investigating anti-inflammatory and antioxidant interventions in pediatric psychosis, especially in subpopulations with higher inflammation and oxidative stress levels at baseline, would add evidence to help determine whether there are causal links between immune and redox dysregulation and psychosis symptom progression in youth. It is important to note that a prior *meta*-analysis found that antipsychotic prescription in adults with schizophrenia reduced plasma levels of proinflammatory cytokines IL-1β and IFN-γ and increased plasma levels of soluble receptor of the proinflammatory cytokine IL-2, providing rationale for the study of antipsychotic intervention in immune biomarker studies (Tourjman et al., 2013).

### 4.4 Limitations

Antipsychotic anti-inflammatory properties may have reduced the immune biomarker effect size differences between psychosis and non-psychosis groups reported in the current *meta*-analyses. The percentage of psychosis spectrum participants on antipsychotics varied greatly between studies, from 0 % (Li et al., 2022; Simsek et al., 2016b; Sporn et al., 2005; Gonzalez-Pinto et al., 2012) to 100 % (Chen et al., 2021). However, the effects of potential confounders like antipsychotic prescription could not be determined for individual biomarkers because there were not enough studies. We also were unable to investigate additional potential confounders based on known biological contributors to peripheral immune marker levels, such as IL-6 production by contracting muscles and TNF production by adipocytes, because of insufficient studies.

Another limitation of the current *meta*-analysis is that there were insufficient studies to examine if psychosis symptom severity and acuity affected biomarker levels. A prior *meta*-analysis found differences in immune activation levels in acutely ill compared to chronically ill patients with schizophrenia, bipolar disorder, and major depressive disorder in adult populations (Goldsmith et al., 2016). An additional limitation of the current *meta*-analysis is the requirement of mean age less than or equal to 18 for inclusion, which excluded the large biomarker study NAPLS, which found that baseline markers of inflammation and oxidative stress were associated with transition from CHR-P to TPD (Perkins et al., 2015). Additionally, immune and redox pathways are related to broad brain functions like cognition, psychomotor slowing, and psychiatric disorders across diagnostic boundaries (Chen et al., 2021; Goldsmith et al., 2020, 2016; Jorgensen et al., 2022; Mittleman et al., 1997; Wang and Miller, 2018), and a limitation of the current *meta*-analysis is the inability to determine the extent that immune and oxidative stress biomarkers are elevated in non-psychotic pediatric psychiatric disorders.

### 4.5. Conclusion

Our *meta*-analysis found that neutrophil/lymphocyte ratio, TNF, CRP, IL-6, and total WBC were elevated in youth with threshold psychosis compared to youth without threshold psychosis, extending results from adult populations. We also reviewed existing studies and gaps in the pediatric literature regarding immune and redox biomarkers in threshold, subthreshold, and familial high-risk for psychosis. Notably, immune and redox biomarker intervention studies are lacking, and research investigating interventions targeting the immune system in threshold pediatric psychosis is especially warranted.

## Supplementary Material

1

2

3

4

5

6

7

## Figures and Tables

**Table 1:** Psychosis-risk and psychosis spectrum definitions.

Psychosis-Risk and Psychosis Spectrum Category	Description

**Threshold Psychotic Disorder (TPD)**	Any psychotic disorder characterized by hallucinations, delusions, and/or disorganized speech that causes functional impairment. A participant was considered to have TPD if: 1. Assessed with standardized interview based on DSM-5 criteria. OR 2. Study participants were diagnosed based on clinician assessment with a DSM-5 (or equivalent diagnosis from earlier DSM version) psychotic disorder, unless the DSM-5 diagnosis was “Attenuated Psychosis Syndrome,” which we categorized as “Subthreshold Psychosis” OR 3. If diagnosed by a clinician assessment as “Other” or “Unspecified” psychotic disorders, and the psychotic symptoms were severe enough to cause hospitalization or occurred for more than an hour, most days of the week, for at least a month and caused functional impairment.
Subtypes of TPD	

Schizophrenia Spectrum (SCZ)	SCZ is a subtype of TPD when the participants were diagnosed with either schizophrenia or schizoaffective disorder. We categorized studies as SCZ in studies where the ultimate diagnosis was schizophrenia or schizoaffective disorder in more than 80 % of cases even when the initial diagnosis at the time of biospecimen collection included “Unspecified” or “Other” psychosis. “Early Onset Schizophrenia” is usually defined as a SCZ onset before age 18. All studies included in our systematic review had a mean age less than 18, making the “Early Onset Schizophrenia” largely redundant with SCZ categorization.
Very Early Onset Schizophrenia Spectrum Disorder (VEOS)	This is a subtype of SCZ with onset of schizophrenia or schizoaffective disorder before age 13. This is also called “Childhood Onset Schizophrenia Spectrum Disorder.”
Affective Psychotic Disorder	Affective psychotic disorder is a subtype of TPD when the participants were diagnosed Bipolar 1 with psychotic features, major depressive disorder with psychotic features, or unspecified mood disorders with psychotic features.
First Episode Psychosis (FEP)	At the time of the study, participants had symptoms for less than two years. This term is often reflective of “recent onset psychosis” because when a first episode of psychosis begins and ends can be challenging to determine. In the literature “recent” or FEP is sometimes defined as symptom onset within the last six months up to the last five years. Two years is commonly used.
Antipsychotic naïve	At the time of study enrollment, participants were excluded if they had ever been previously treated with an antipsychotic.
**Subthreshold Psychosis (SP)**	
Clinical High Risk for Psychosis (CHR-P)	Participants are considered CHR-P if psychosis symptoms meet criteria based on the Structured Interview for Psychosis-Risk Syndromes (SIPS) (Miller et al., 1999) or the Comprehensive Assessment of At Risk Mental States (CAARMS) (Yung et al., 2005). CHR-P studies are sometimes called “Ultra High Risk for Psychosis” studies, particularly when the CAARMS is used.
Psychotic Like Experiences (PLE)	Studies were categorized as PLE if perceptual disturbances, odd beliefs, and other unusual experiences of the psychosis spectrum were quantified or categorized based on screening measures briefer than SIPS and CAARMS.
**Familial High Risk for Psychosis (FHR-P)**	Participants were FHR-P if they had relatives with a TPD as defined by the study. While most individuals with FHR-P do not develop a TPD, FHR-P is a genetic and potentially environmental risk factor for TPD.

## Data Availability

Data will be made available on request.
