# Supplementary figures and images for: Immune and oxidative stress biomarkers in pediatric psychosis and psychosis-risk: Meta-analyses and systematic review
Source: Brain Behav Immun. Author manuscript; Available in PMC 2025 Mar 1. (PMC10932921; doi:10.1016/j.bbi.2023.12.019)

Supplementary Figure 1a. Effect sizes for biomarkers of immune activation

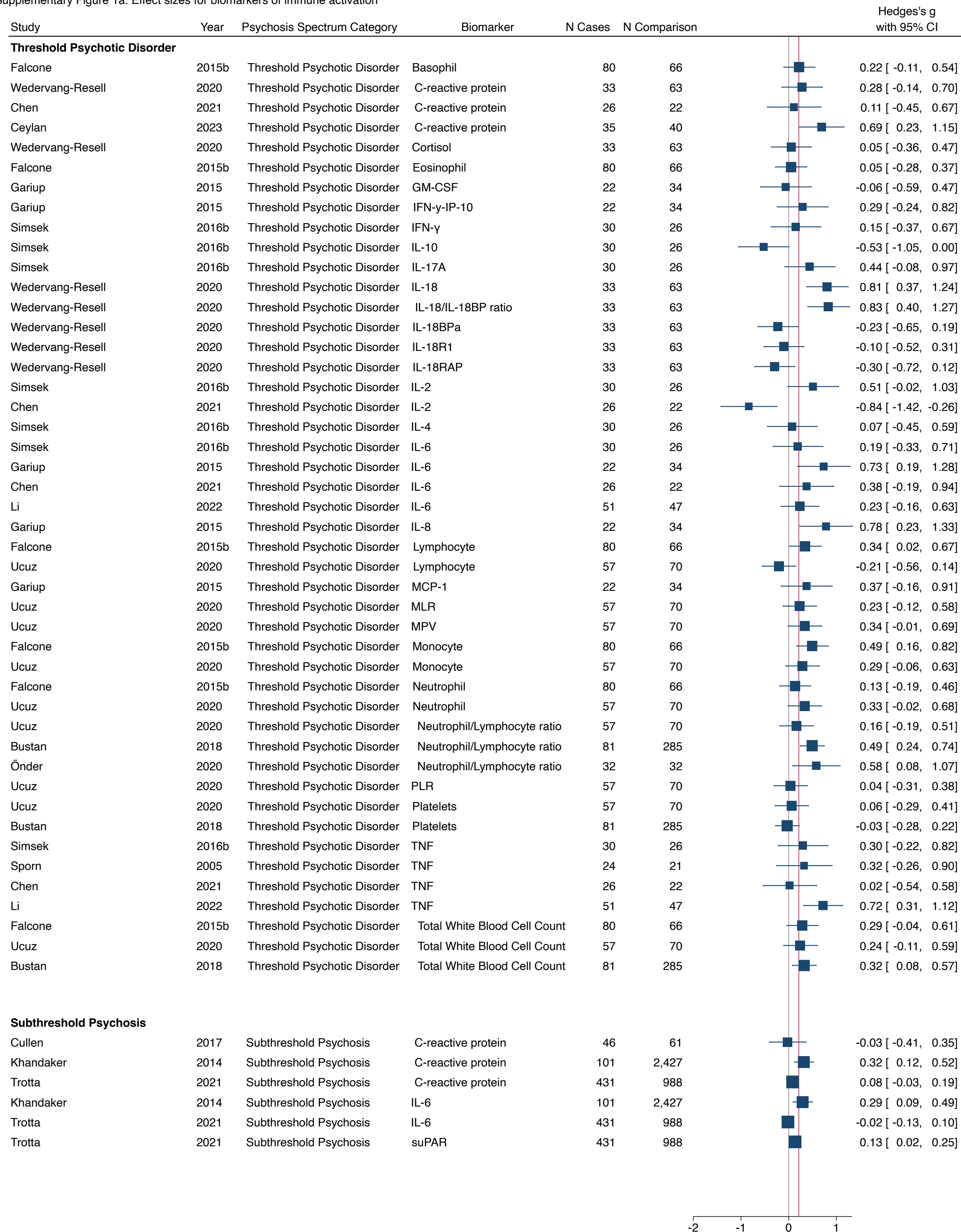

Supplement: 1 [file NIHMS1958543-supplement-1.pdf]

Supplementary Figure 1d. Effect sizes for other biomarkers

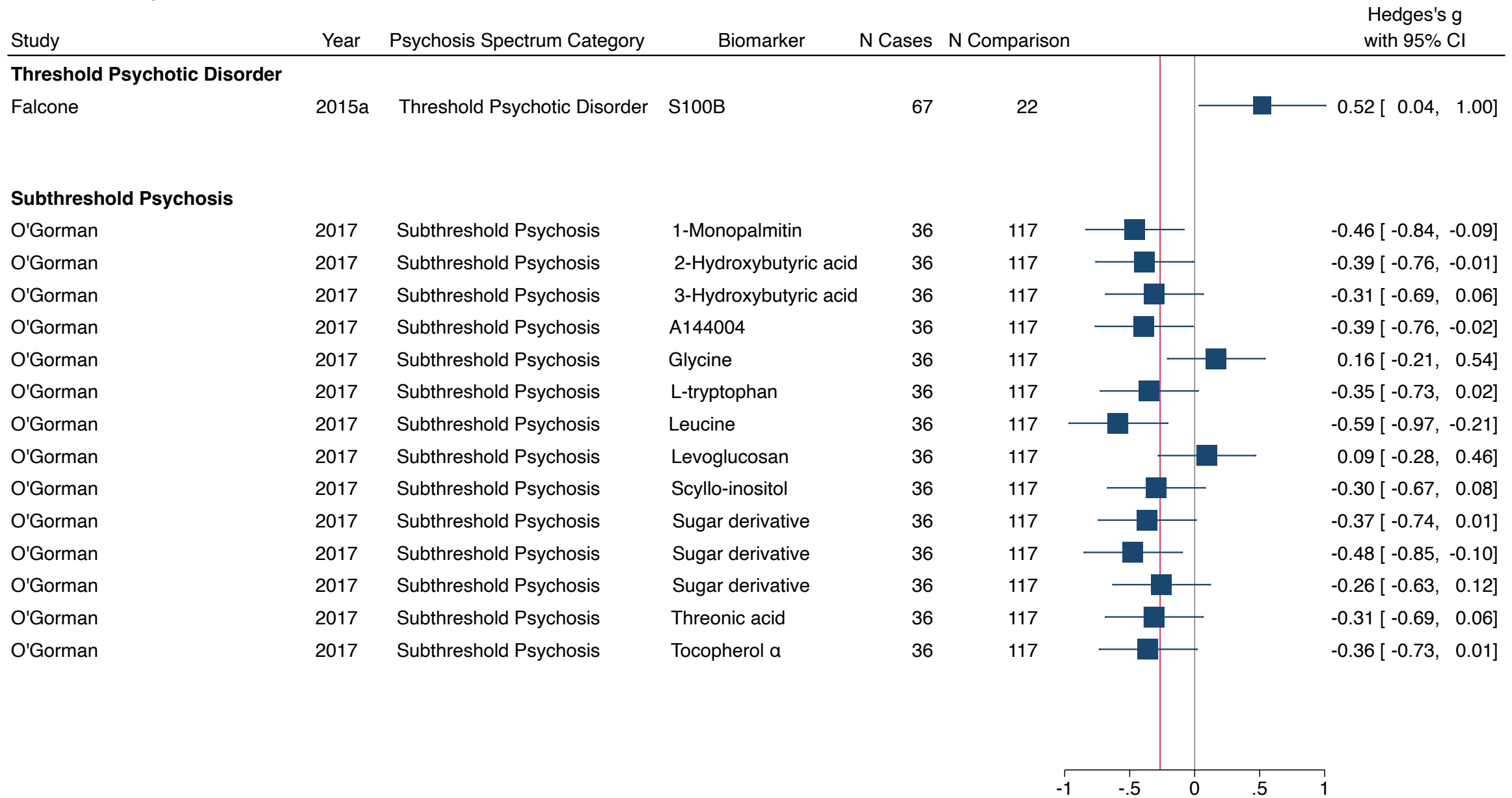

Random-effects REML model

Supplement: 4 [file NIHMS1958543-supplement-4.pdf]

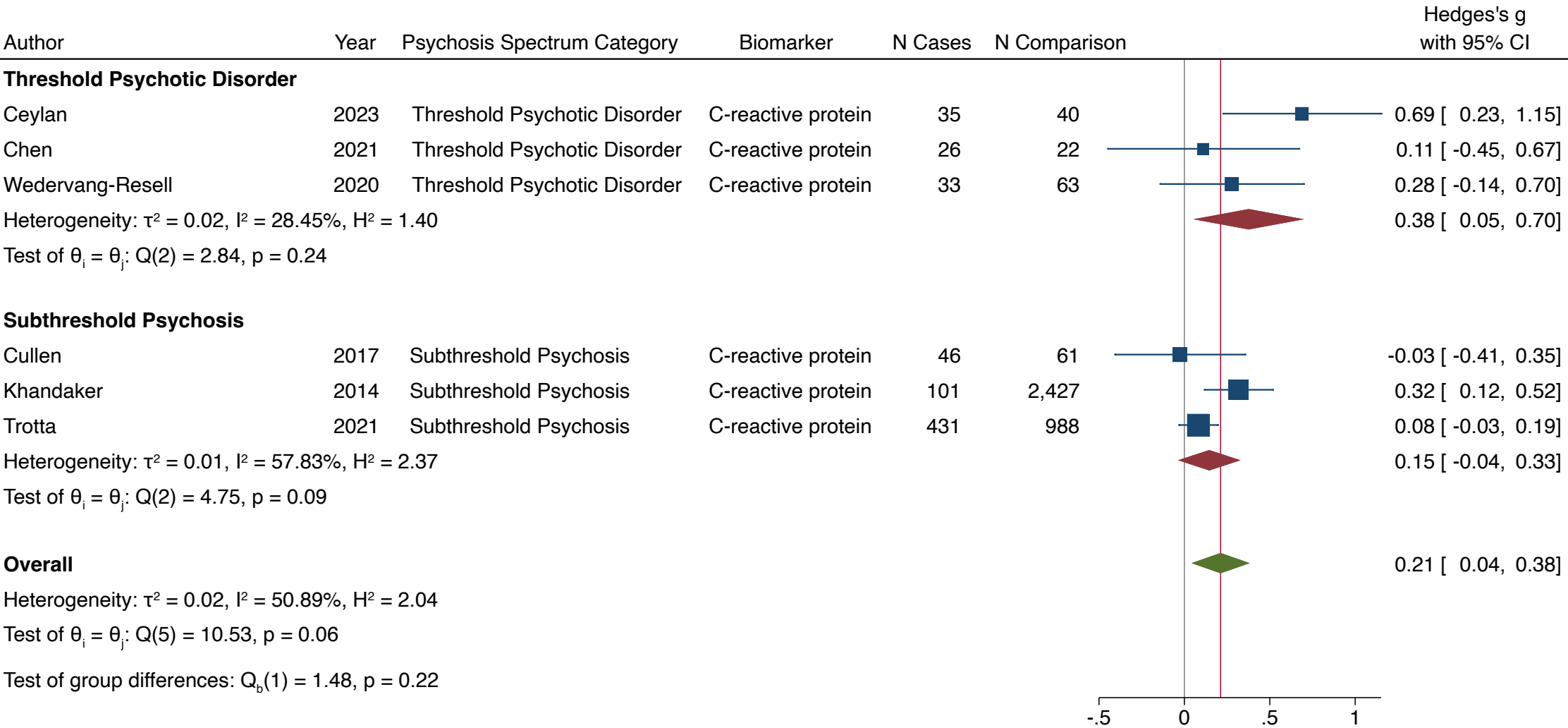

Supplement: 5 [file NIHMS1958543-supplement-5.pdf]
