# Supplementary material for: Immune and oxidative stress biomarkers in pediatric psychosis and psychosis-risk: Meta-analyses and systematic review
Source: Brain Behav Immun. Author manuscript; Available in PMC 2025 Mar 1. (PMC10932921; doi:10.1016/j.bbi.2023.12.019)
Supplement: 2 [file NIHMS1958543-supplement-2.pdf]

pplementary Figure 1b. Effect sizes for biomarkers of oxidative stress

Study	Year	Psychosis Spectrum Category	Biomarker	N Cases	N Comparison	Hedges's g with 95% CI
**Threshold Psychotic Disorder**						
Mico	2011	Threshold Psychotic Disorder	Catalase	102	95	-0.05 [-0.33, 0.23]
Parellada	2012	Threshold Psychotic Disorder	Catalase	34	34	-0.37 [-0.84, 0.11]
Simsek	2016a	Threshold Psychotic Disorder	CoQ	20	20	-0.05 [-0.66, 0.55]
Mico	2011	Threshold Psychotic Disorder	Glutathione	102	95	-4.72 [-5.26, -4.17]
Parellada	2012	Threshold Psychotic Disorder	Glutathione	34	34	0.05 [-0.42, 0.52]
Mico	2011	Threshold Psychotic Disorder	Glutathione Peroxidase	102	95	2.10 [1.76, 2.45]
Simsek	2016a	Threshold Psychotic Disorder	Glutathione Peroxidase	20	20	0.09 [-0.52, 0.70]
Parellada	2012	Threshold Psychotic Disorder	Glutathione Peroxidase	34	34	-0.63 [-1.11, -0.15]
Parellada	2012	Threshold Psychotic Disorder	Homocysteine	34	34	1.54 [1.00, 2.07]
Mico	2011	Threshold Psychotic Disorder	Lipid Hydroperoxides	102	95	3.22 [2.79, 3.64]
Mico	2011	Threshold Psychotic Disorder	Superoxide Dismutase	102	95	0.34 [0.06, 0.62]
Simsek	2016a	Threshold Psychotic Disorder	Superoxide Dismutase	20	20	-0.24 [-0.85, 0.37]
Parellada	2012	Threshold Psychotic Disorder	Superoxide Dismutase	34	34	-0.55 [-1.03, -0.07]
Mico	2011	Threshold Psychotic Disorder	TAS	102	95	-1.21 [-1.52, -0.91]
Parellada	2012	Threshold Psychotic Disorder	TAS	34	34	0.07 [-0.40, 0.54]
Parellada	2012	Threshold Psychotic Disorder	Thiobarbituric acid reactive substances;	34	34	0.06 [-0.41, 0.53]
Lundberg	2022	Threshold Psychotic Disorder	Trx1	23	20	-0.01 [-0.60, 0.57]
**Subthreshold Psychosis**						
Wake	2022	Subthreshold Psychosis	8OHdG/Cre	19	21	-0.14 [-0.75, 0.47]
Wake	2022	Subthreshold Psychosis	Biopyrrin/Cre	19	21	0.91 [0.27, 1.55]
Wake	2022	Subthreshold Psychosis	Biopyrrin/FLC	19	21	1.11 [0.45, 1.76]
Wake	2022	Subthreshold Psychosis	CRP/Cre	19	21	-0.20 [-0.81, 0.41]
Wake	2022	Subthreshold Psychosis	CX3CL1/Cre	19	21	-0.26 [-0.87, 0.35]
O'Gorman	2017	Subthreshold Psychosis	Citric acid	36	117	-0.48 [-0.85, -0.10]
Wake	2022	Subthreshold Psychosis	Cortisol/Cre	19	21	0.66 [0.03, 1.28]
Wake	2022	Subthreshold Psychosis	FLC/Cre	19	21	-4.17 [-5.26, -3.07]
Wake	2022	Subthreshold Psychosis	Kflc/lFLC ratio	19	21	-2.05 [-2.81, -1.29]
O'Gorman	2017	Subthreshold Psychosis	Oleic acid	36	117	-0.28 [-0.65, 0.09]
O'Gorman	2017	Subthreshold Psychosis	Palmitic acid	36	117	-0.37 [-0.75, 0.00]
O'Gorman	2017	Subthreshold Psychosis	Stearic acid	36	117	-0.37 [-0.75, 0.00]
**Familial High Risk for Psychosis**						
Gonzalez-Pinto	2012	Familial High Risk for Psychosis	Catalase	14	82	-0.40 [-0.96, 0.17]
Gonzalez-Pinto	2012	Familial High Risk for Psychosis	Glutathione	14	82	0.31 [-0.25, 0.88]
Gonzalez-Pinto	2012	Familial High Risk for Psychosis	Glutathione Peroxidase	14	82	0.08 [-0.48, 0.64]
Gonzalez-Pinto	2012	Familial High Risk for Psychosis	Lipid Hydroperoxides	14	82	0.35 [-0.21, 0.92]
Gonzalez-Pinto	2012	Familial High Risk for Psychosis	Superoxide Dismutase	14	82	-0.49 [-1.05, 0.08]
Gonzalez-Pinto	2012	Familial High Risk for Psychosis	TAS	14	82	-0.77 [-1.35, -0.20]

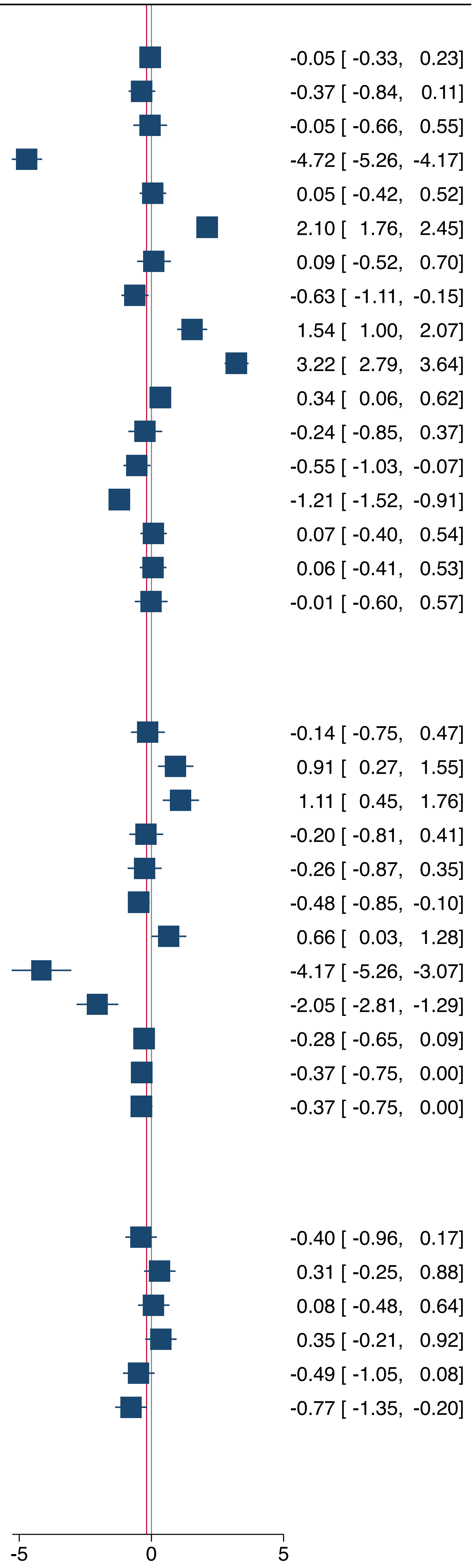

Random-effects REML model
