# Supplementary material for: Immune and oxidative stress biomarkers in pediatric psychosis and psychosis-risk: Meta-analyses and systematic review
Source: Brain Behav Immun. Author manuscript; Available in PMC 2025 Mar 1. (PMC10932921; doi:10.1016/j.bbi.2023.12.019)
Supplement: 3 [file NIHMS1958543-supplement-3.pdf]

Supplementary Figure 1c. Effect sizes for biomarkers of bioactive lipids

Study	Year	Psychosis Spectrum Category	Biomarker	N Cases	N Comparison	Hedges's g with 95% CI
**Threshold Psychotic Disorder**						
Wedervang-Resell	2020	Threshold Psychotic Disorder	TC/HDL-C ratio	33	63	0.85 [0.42, 1.29]
Wedervang-Resell	2020	Threshold Psychotic Disorder	TG	33	63	0.78 [0.35, 1.22]
**Subthreshold Psychosis**						
O'Gorman	2017	Subthreshold Psychosis	Cholesterol	36	117	-0.30 [-0.67, 0.07]
O'Gorman	2017	Subthreshold Psychosis	Lysophosphatidylcholine(16:0)	36	117	-0.38 [-0.76, -0.01]
O'Gorman	2017	Subthreshold Psychosis	Lysophosphatidylcholine(16:1)	36	117	-0.43 [-0.81, -0.06]
O'Gorman	2017	Subthreshold Psychosis	Lysophosphatidylcholine(22:6)	36	117	-0.63 [-1.01, -0.25]
O'Gorman	2017	Subthreshold Psychosis	Phosphatidylcholine(31:0)	36	117	-0.46 [-0.83, -0.08]
O'Gorman	2017	Subthreshold Psychosis	Phosphatidylcholine(32:1)	36	117	-0.24 [-0.62, 0.13]
O'Gorman	2017	Subthreshold Psychosis	Phosphatidylcholine(33:1)	36	117	-0.49 [-0.87, -0.11]
O'Gorman	2017	Subthreshold Psychosis	Phosphatidylcholine(35:1)	36	117	-0.37 [-0.74, 0.00]
O'Gorman	2017	Subthreshold Psychosis	Phosphatidylcholine(36:2)	36	117	-0.25 [-0.62, 0.13]
O'Gorman	2017	Subthreshold Psychosis	Phosphatidylcholine(36:4)	36	117	0.02 [-0.35, 0.40]
O'Gorman	2017	Subthreshold Psychosis	Phosphatidylcholine(37:4)	36	117	-0.30 [-0.68, 0.07]
O'Gorman	2017	Subthreshold Psychosis	Phosphatidylcholine(38:6)	36	117	-0.12 [-0.50, 0.25]
O'Gorman	2017	Subthreshold Psychosis	Phosphatidylcholine(40:6)	36	117	-0.23 [-0.60, 0.15]
O'Gorman	2017	Subthreshold Psychosis	Phosphatidylcholine(40:7)	36	117	-0.26 [-0.63, 0.11]
O'Gorman	2017	Subthreshold Psychosis	Phosphatidylcholine(O-38:4)	36	117	-0.44 [-0.81, -0.06]
O'Gorman	2017	Subthreshold Psychosis	Phosphatidylcholine(O-40:6)	36	117	-0.40 [-0.77, -0.02]
O'Gorman	2017	Subthreshold Psychosis	Phosphatidylcholine(O-42:3)	36	117	-0.40 [-0.77, -0.03]
O'Gorman	2017	Subthreshold Psychosis	Phosphatidylethanolamines (P-18:0/22:6)	36	117	-0.37 [-0.75, -0.00]
O'Gorman	2017	Subthreshold Psychosis	Sphingomyelin(D18:0/16:0)	36	117	-0.20 [-0.57, 0.18]
O'Gorman	2017	Subthreshold Psychosis	Sphingomyelin(D33:1)	36	117	-0.35 [-0.73, 0.02]
O'Gorman	2017	Subthreshold Psychosis	Triacylglycerol(49:3)	36	117	-0.37 [-0.74, 0.01]
O'Gorman	2017	Subthreshold Psychosis	Triacylglycerol(51:2)	36	117	-0.54 [-0.91, -0.16]
O'Gorman	2017	Subthreshold Psychosis	Triacylglycerol(56:7)	36	117	-0.16 [-0.53, 0.21]

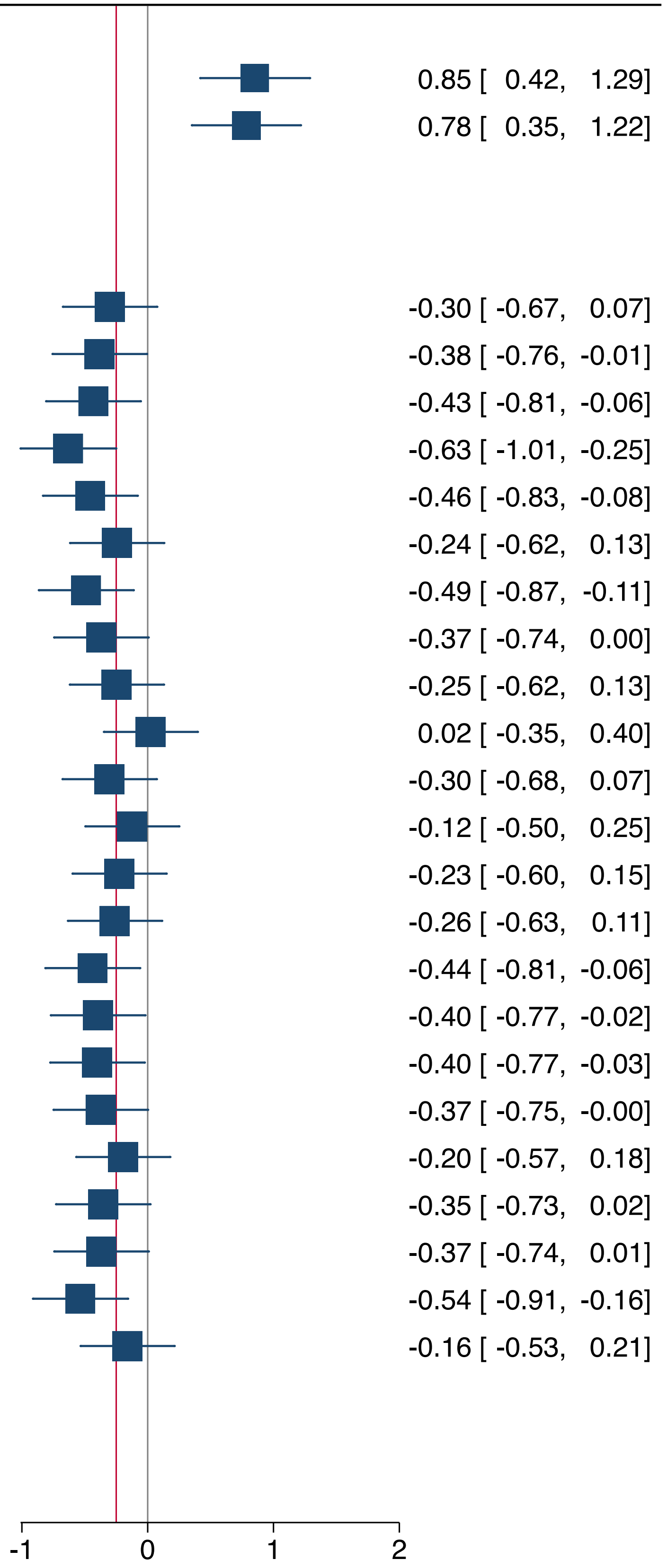

Random-effects REML model
