# Supplementary material for: Immune and oxidative stress biomarkers in pediatric psychosis and psychosis-risk: Meta-analyses and systematic review
Source: Brain Behav Immun. Author manuscript; Available in PMC 2025 Mar 1. (PMC10932921; doi:10.1016/j.bbi.2023.12.019)
Supplement: 6 [file NIHMS1958543-supplement-6.docx]

**Supplementary Table 5**. Meta-regression

| Results of Meta-regression analysis: Immune Activation Biomarkers | | | | |
| --- | --- | --- | --- | --- |
| Variable | β | SE | 95% CI | p |
| % of males | $.0040478$ | $.005185$ | $-.0061145-(.0142102)$ | $0.435$ |
| Body mass index | $-.0473346$ | $.0474708$ | $-.1403757-(.0457064)$ | $0.319$ |
| Age | $-.0183416$ | $.0387386$ | $-.0942678-(.0575846)$ | $0.636$ |
| % of smokers* | $-.0041792$ | $.0159357$ | $-.0354126-(.0270541)$ | $0.793$ |
| % on antipsychotic medication | $-.0022585$ | $.0019139$ | $-.0060096-(.0014927)$ | $0.238$ |
| Subthreshold psychosis | $-.0972801$ | $.0951358$ | $-.2837428-(.0891826)$ | $0.307$ |
| Quality assessment score | $-.0022868$ | $.0363746$ | $-.0690061-(.0735797)$ | $0.950$ |

| Results of Meta-regression analysis: Oxidative Stress Biomarkers | | | | |
| --- | --- | --- | --- | --- |
| Variable | β | SE | 95% CI | p |
| % of males | $.0018968$ | $.0362922$ | $-.0692346-(.0730282)$ | $0.958$ |
| Age | $.035988$ | $.6783579$ | $-1.293569-(1.365545)$ | $0.958$ |
| % on antipsychotic medication | $-.0033984$ | $.0525423$ | $-.1063793-(.0995825)$ | $0.948$ |
| Subthreshold psychosis | $-.4198701$ | $.5279033$ | $-1.454541-(.6148013)$ | $0.728$ |
| Quality assessment score | $.0313143$ | $.4515101$ | $-.8536292-(.9162578)$ | $0.945$ |
| *≤10 observations | | | | |
