# Supplementary material for: Immune and oxidative stress biomarkers in pediatric psychosis and psychosis-risk: Meta-analyses and systematic review
Source: Brain Behav Immun. Author manuscript; Available in PMC 2025 Mar 1. (PMC10932921; doi:10.1016/j.bbi.2023.12.019)
Supplement: 7 [file NIHMS1958543-supplement-7.docx]

**Supplementary Materials**

**Title:** Immune and oxidative stress biomarkers in pediatric psychosis and psychosis-risk: Meta-analyses and systematic review

**Table of Contents**

[Supplementary Methods. Supplementary search strategies 2](#_Toc147688160)

[Supplementary Table 1. Characteristics and findings from reviewed studies. 3](#_Toc147688161)

[Supplementary Table 2. Studies that investigate each biomarker (immune, oxidative stress, bioactive lipids, other) 18](#_Toc147688162)

[Supplementary Table 2a. Immune Activation 18](#_Toc147688163)

[Supplementary Table 2b. Biomarkers of Oxidative Stress 20](#_Toc147688164)

[Supplementary Table 2c. Bioactive Lipids including Fatty Acids and Phospholipids 21](#_Toc147688165)

[SupplementaryTable 2d. Other Biomarkers 21](#_Toc147688166)

[Supplementary Table 3. Biosample collection, storage, and analysis descriptions 23](#_Toc147688167)

[Supplementary Figure 2. Funnel plots for threshold psychosis 29](#_Toc147688168)

[Supplementary Table 4. Quality assessment scores by study 36](#_Toc147688169)

**Supplementary Figure 1, Figure 3, and Table 5 are in separate supplementary files.**

# **Supplementary Methods.** Supplementary search strategies

- PubMed/MEDLINE: (schizophrenia OR psychosis OR psychotic) AND (complement OR oxidative OR inflammation OR "inflamm" OR "inflammatory" OR "anti-inflammatory" OR "cytokine" OR "interleukin" OR "interferon" OR "tumor necrosis factor" OR "oxidat" OR "oxidation" OR "oxidative") AND (children OR adolescent OR adolescence OR youth OR youths OR pediatric)
- Web of Science Core Collection search: TS=((*schizoph* OR "psychosis" OR "psychotic") AND ("complement" OR *oxidat* OR *inflamm* OR "cytokine" OR "interleukin" OR "interferon" OR "tumor necrosis factor") AND (children OR *adolescen* OR *youth* OR pediatric)

Excluded: (MEETING OR RETRACTED PUBLICATION OR BOOK OR REFERENCE MATERIAL OR EDITORIAL OR LETTER OR NEWS OR RETRACTION)

No date limits were applied and included results from database inception through June 1, 2022.

**Supplementary Table 1. Characteristics and findings from reviewed studies.** Hedge’s *g* effect sizes were caclulated when sufficient data for psychosis and non-psychosis groups were available. Bolded studies were included in the meta-analyses.

| **Study** | **Study type, cohort if applicable** | **Country** | **Psychosis spectrum type** | **Case Definition** | **Comparison group definition** | **Sample size (n) (% identified female)** | | **Age M±SD** | | | **Race,**  **Ethnicity (%)** | **Biomarkers findings (effect sizes when available) and other variables measured (BMI, smoking, substance use, medication)** |
| --- | --- | --- | --- | --- | --- | --- | --- | --- | --- | --- | --- | --- |
|  |  |  |  |  |  | Psychosis spectrum | Comparison | Psychosis spectrum | | Comparison |  |  |
| Amminger et al. (2010) | RCT | Austria | SP (CHR-P), antipsychotic-free | UHR based on PANSS assessment | N/A | 80 (65% F) | N/A | 16.9±2.42 | |  | Not reported | 12 week intervention of 1.2g omega-3 PUFAs reduced positive, negative, & general symtoms as compaired to placdebo.  Other markers measured in whole blood: RBC membrane A-Linolenic acid (parent fatty acid (FA) to omega-3 FA), Eicosapentaenoic acid, Docosapentaenoic acid, Docoshexaenoic acid, Linoleic acid, Arachidonic acid  Measured variables included: BMI, tobacco use, marijuana use, antidepressant medication |
| Berger et al. (2016) | Transition to TPD; Treated arm of Amminger et al. (2010) RCT | Austria | SP (CHR-P) progression to TPD, antipsychotic-free | Semi-structured interview using UHR criteria, PANSS, DSM-IV criteria for SCZ/schizophreniform for TPD determination | CHR-P that did not progress to TPD | 11 (progresed to TPD), 66.7% F | 54 (remained SP), 40% F | 16.4±1.3 | | 16.5±2.3 | Excluded patients with “Non-Caucasian skin type” | Niacin sensitivity increased in CHR-P compared to HC, attenuated in FEP compared to HC. In CHR-P niacin sensitivity inversely correlated w/ omega-6 & 9 fatty acids.  Other markers measured in plasma: RBC membrane fatty acids  Measured variables included: nicotine use, marijuana use, drugs other than THC, antipsychotic medication, antidepressant medication |
| Berger et al. (2018) | Cohort recruited from negative fish oil randomized-controlled trial; NEURAPRO | Australia | SP (CHR-P), antipsychotic-free | CAARMS | N/A | 106, 66.04% F | N/A | 17.21±2.37 | | | Not reported | Used biomarkers and markers of allostatic load (BMI, blood pressure, heart rate lipids) to create allostatic index, which predicted poorer functioning and more manic symtpoms at 6 months but not 12 months.  Markers measured in plasma: CRP, IL-6, IL-12. Markers measured in saliva: cortisol.  Measured variables included: BMI, smoking. |
| **Bustan** et al. (2018) | Case-control | Israel | TPD (SCZ) | Positive and disorganized psychosis symptoms in EMR. 87.7% diagnosed with SCZ | Admitted to the psychiatric inpatient unit without an affective or psychotic disorder | 81, 42% F | 285, 48.4% F | 15.9±1.6 | | 14.7±1.8 | Not reported | Calculated Hedge’s g (95% CI):  Total WBC 0.32 (0.08, 0.57)  Platelets –0.03 (-0.28, 0.22)  Neutrophil/Lymphocyte ratio 0.49 (0.24, 0.74)  Measured variables included: BMI, smoking status, antipsychotic medication at admission |
| **Ceylan** et al. (2023) | Case-control | Turkey | TPD (SCZ) | DSM-5 SCZ diagnosis; K-SADS-PL | Healthy comparison subjects with similar age and gender, with sociodemographic data & clinical features reported | 25, 45.7% F | 40, 40% F | Median 16.00, IQR 2 | | Median 16.00, IQR 2 | Not reported | SCZ: serum median CRP 2.3 (IQR 3.7)  Control: serum median CRP 0.5 (IQR 0.75)  CRP units not defined in manuscript |
| **Chen** et al. (2021) | Case-control | Taiwan | TPD (SCZ with FEP) | DSM-5 SCZ diagnosis with first episode of illness, Clinical Global Impression-Severity scale for Schizophrenia score ≤ 3 | Age and sex-matched controls with no psychiatric symptoms based on Mini-International Neuropsychiatric Interview | 26, 57.7% F | 22, 63.6% F | 17.92 ±2.59 | | 17±1.2 | Not reported | Calculated Hedge’s g (95% CI): CRP 0.11 (-0.45, 0.67); IL-2 –0.84 (-1.42, -0.26); IL-6 0.38 (-0.19, 0.94); TNF 0.02 (-0.54, 0.58)  Measured variables included: BMI, and symptom scores |
| **Cullen** et al. (2017) | Case-control | UK | SP (PLE), antipsychotic-free | Five-item questionnaire adapted from Diagnostic Interview Schedule for Children (DIS-C)- PLEs present | PLEs absent | 46, whole sample 51.9% F | 61, whole sample 51.9% F | Whole sample 13.2±1.1 | | | White British (32.7), White other (26.2), Black African or Caribbean (11.2), Other (29.9) | Calculated Hedge’s g (95% CI):  Salivary CRP -0.03 (-0.41, 0.35)  Measured variables included: BMI, tobacco, cannabis use |
| English et al (2018) | Case-control; ALSPAC | UK | SP (PLE) | PLIKS interview, symptoms at least once a month over 6 months with distress or impairment. | PLEs absent | Discovery n = 37; Verification n = 40; 78.37% F | Discovery n = 38; Verification n = 66 | Plasma collected at age 11 (SD unavailable), Psychosis symptoms assesed at age 18 (SD unavailable) | | | White (91.2), Non-white (2.2), N/A (6.6) | Complement differences at age 11 were associated with PLEs at age 18 using proteomics.  Proteomic profiling completed using plasma samples  Measured variables included: BMI |
| Falcone et al. (2015a) | Case-control | USA | TPD (FEP) | Semi-structured interview developed using DSM-IV criteria for psychotic disorder | Healthy controls | 67, whole sample 45.1% F | 22 | 16.59 ±1.01 | | 14.6 ±2.42 | Not reported | Calculated Hedge’s g (95% CI):  S100B 0.52 (0.04, 1.00)  Measured variables included: psychotropic medication |
| **Falcone** et al. (2015b) | Case-control | USA | TPD (FEP) | DSM-IV-TR diagnosis of Psychosis NOS, Schizophreniform, or SCZ in EMR | Inpatients without psychosis | 80, 37.5% F | 66, 45.5% F | 13.6±2.89 | | 13.7 ±2.8 | White (55.5), Non-Caucasian (44.5) | Calculated Hedge’s g (95% CI):  Basophil 0.22 (-0.11, 0.54);  Eosinophil 0.05 (-0.28, 0.37);  Lymphocyte 0.34 (0.02, 0.67);  Monocyte 0.49 (0.16, 0.82);  Neutrophil 0.13 (-0.19, 0.46);  Total WBC 0.29 (-0.04, 0.61)  Cases and controls were matched for age, race, gender, substance use, medication, BMI |
| Föcking et al. (2016) | Case-control; Placebo arm of Amminger et al. (2010) RCT | Ireland | TPD (FEP) | UHR - inclusion criteria proposed by Yung that received placebo and progressed to psychosis | Participants at risk who did not progress to a first episode of psychosis | 11, 63.3% F | 28, 71.42% F | 15.88 ±0.33 | | 16.2 ±0.34 | Not reported | Baseline plasma levels of the inflammatory marker IL-12-IL23-p40 are associated with transition from SP to TPD. IL12/IL23p40 levels did not change following 12 weeks administration of omega-3 PUFAs.  Other markers measured in plasma: CRP, Eotaxin, Eotaxin-3, IFN-γ, IL-10, IL-12-IL-23-p40, IL-15, IL-16, IL-17, IL-1α, IL-1β, IL-5, IL-6, IL-7, IL-8, IP-10, MCP-1, MCP-4, MDC, MIP-1α, MIP-1β, PIGF, SAA, TARC, TNF-α, Lymphotoxina alphia (aka TNF-β), Tie-2, VEGF, VEGF-C, VEGF-D, bFGF, sFlt-1, sICAM-1, sVCAM-1  Measured variables included: BMI, smoking status (yes/no), alcohol use, marijuana use, antidepressant use, benzodiazepine use |
| Föcking et al. (2021) | Case-control; ALSPAC | England | SP (PLE) | PLIKS Interview, subjects who fulfilled criteria for definite PEs at age 18 but not age 12 | Age-matched controls randomly selected from individuals within ALSPAC cohort with available plasma samples at age 12 and no PLE’s at ages 12 or 18 | 64, 56.25% F | 67, 41.79% F | Biospecimens at age ~12 (SD unavailable) for cases & controls; psychosis symptoms at age 18 (SD unavailable) | | | White (92.36), Non-white (2.3), N/A (5.34) | Higher plasma levels of several complement proteins at age 12 were associated with subsequent PLEs at age 18 in a proteomic analysis  Other markers measured in plasma: Vitronectin, Complement C1r, Subcomponent-like protein, Complement componenet C8 beta chain, complement component C8 alpha chain, Complement factor H, Complement C5, C4b-binding protein alpha chain, Complement C2, Mannan-binding lectin serine protease 1, Complement C1s subcomponent, Complement factor B, Complement component C8 gamma chain, Complement C1q subcomponent subunit A, Complement C4-A, Complement C1q subcomponenet subunit B, Complement factor 1, Clusterin, Complement C1r subcomponent, Compliment component C6, Compement C1q subcomponent subunit C, C4b-binding protein beta chain, Complement factor H-related protein 5, Complement component C9, Complement factor D, Plasma protease, C1 inhibitor, Mannose-binding protein C, Complement C4-B, Complement component C7, Complement C3  Measured variables included: BMI |
| Garcia et al. (2018) | Cohort (with cross-sectional analysis at baseline, 1 year, and 2 year timepoints); CAFEPS | Spain | TPD (FEP) | K-SADS-PL at admission; DSM-IV criteria at follow-up | N/A | Whole sample 70, 28.6% F | N/A | Whole sample baseline age: 15.7±1.63 | | | Caucasian (91.4), Hispanic (5.7), Other (2.9) | At baseline, higher plasma TAS was associated with better outcomes (higher functioning on CGAS) but not associated with symptoms on PANSS or YMRS. However, longitudinal linear models found relationships between higher plasma TAS and poorer outcomes: CGAS (β = -0.006, p = 0.004, 95% CI: (-0.010, -  0.002)), PANSS pos (β = 0.013, p = 0.040, 95% CI: (0.001, 0.025)) and YMRS scales (β = 0.014, p = 0.007, 95% CI: (0.004, 0.025)) after adjusting for sex.  Markers measured in plasma: TAS  Measured variables included: Age, sex, ethnic group, parental level of education, socioeconomic level, living arrangements, use of toxic substances and dose of medication in chlorpromazine equivalent units |
| **Gariup** et al. (2015) | Case-control | Spain | TPD | K-SADS-PL * with DSM-IV criteria | Healthy community subjects within the same age range, without history of psychiatric diagnosis, nor current according to the semi-structured interview (K-SADS-PL), except for ADHD | 22, %F unavailable for this diagnostic subset | 34, 59% F | 15.6±2.67 | | 16.5±2.3 | Not reported | IL-1β̞, IL-6, IL-8, IL-10 , monocyte chemoattractant protein-1 and monocytes significantly higher in TPD. Confirmed when adjusted for BMI, age, gender, and drug intake at admission. Significant correlation b/t stress and some inflammatory markers.  Other markers measured in whole blood & serum:  GM-CSF -0.06 (-0.59, 0.47)  IFN-γ 0.29 (-0.24, 0.82)  IL-1b  IL-2  IL-4  IL-5  IL-6 0.73 (0.19, 1.28)  IL-8 0.78 (0.23, 1.33)  IL-10  TNF-α  IP-10  MCP-1 0.37 (-0.16, 0.91)  Monocytes, WBC differential  *Only IL-6 had median/IQR data for psychosis that could be used in meta-analysis.  Measured variables included: BMI, age, gender, THC present at admission, child and parent stress measures, family integrity |
| **Gonzalez-Pinto** et al. (2012) | Case-control; CAFEPS | Spain | FHR-P, antipsychotic-free | Healthy controls with second-degree relatives with psychotic disorder by DSM-IV criteria. | Healthy controls without a family history of psychotic illness | 14, 42.86% F | 82, 36.59% F | 15.86 ±1.23 | | 15.06 ±2.03 | Not reported | TAS significantly higher in healthy controls compared to healthy controls with family history of psychosis  Other markers measured in plasma: TAS, glutathione, lipid hydroperoxides, catalase, superoxide dismutase, glutathione peroxidase  Measured variables included: tobacco use |
| **Khandaker** et al. (2014a) | Cohort; ALSPAC | England | SP (PLE) | PLIKS interview | PLEs absent | 101, whole sample 49% F | 2427 | Biospecimens collected at age 9 (SD unavailable), psychosis symptoms measured at age 17.8 (0.38) | | | White British (98.0), Non-white (2.0) | Calculated Hedge’s g (95% CI): Serum  IL-6 0.29 (0.09, 0.49);  CRP 0.32 (0.12, 0.52)  Measured variables included: BMI  BMI (higher serum IL-6 levels at age 9 associated with higher BMI (p <0.001)) |
| Khandaker et al. (2021) | Cohort; ALSPAC | England | SP (PLE) | 10-item questionnaire based on PLIKS interview; cases defined as definitely experiencing a psychotic symptom | PLEs absent | 3488, 51.29% F |  | Serum CRP age ~16 (Whole sample 16.6±2.82); psychosis symptoms age ~17 (SD unavailable) | | | White British (89.6), other (10.4) | CRP associated with auditory hallucinations and anhedonia. Inflammation associated with sub-clinical psychotic symptoms in young people in general population.  Markers measured in serum: CRP  Measured variables included: BMI, tobacco, cannabis, other drug use |
| **Li** et al. (2022) | Case-control | China | TPD (SCZ, FEP, antipsychotic-free) | Structured clinical interview, met DSM-IV criteria for SCZ | Healthy controls, no current or past illness history of Axis I disorders | 51, 56.9% F | 47, 55.32% F | 17(IQR =2) | | 19 (IQR=6) | Not reported | Calculated Hedge’s g (95% CI):  IL-6 0.23 (-0.16, 0.63)  TNF 0.72 (0.31, 1.12)  Measured variables included: BMI |
| Lizano et al. (2016) | Case-control | USA | FHR-P with TPD compared to FHR-P without TPD | FHR-P (first or second degree relative) with no TPD at baseline but progressed to TPD at 3-year follow-up on DSM-IV SCID | FHR-P without progression to TPD over 3 years | 3, whole sample 57.15% F | 32 | Serum collected at baseline age 16.5±0.6 (Up to 3-year follow-up for clinical progression to TPD) | | | Caucasian (51.3), African-American (39.5), Asian (5.9), Hispanic (1.6), Other (1.6) | The 3 converters to psychosis in the FHR group had higher mean values for IL-10, IL-12, PlGF, VEGF, bFGF, and sFlt-1 compared to FHR-P.  Markers measured IFN-γ, IL-1β, IL-6, IL-8, IL-10, IL-12, TNF, PIGF, VEGF, BDNF, bFGF, sFlt-1 |
| Lundberg et al. (2022) | Case-control | Sweden | TPD (EOP) | Early onset psychosis syndromes, diagnosis established by board-certified psychiatrists | Healthy age-matched controls, no disorders on K-SADS-PL | 23, 56.6% F | 20, 75% F | 17.1 | 15.5 | | Not reported | No significant difference (p>0.7) in plasma Trx1 levels between cases & controls was observed.  Case (mean±SEM): Trx1 18.1±7.7; TGF-b 6.4±3.0  Control (mean±SEM): Trx1 18.6±6.3; TGF-b 6.2±2.7 |
| Madrid-Gambin et al. (2019) | Case-control; ALSPAC | England | SP (PLE) | PLIKS interview | No PLEs according to PLIKS | 48, 54.16% F | 67, 41.79% F | Biomarkers at age 12 (SD unavailable), PLEs at age 18 (SD unavailable) | | | From ALSPAC baseline data: White (96.09) | Lipidome & proteome of subjects with PLEs at 18 years of age are already altered at 12 years of age, indicating metabolic dysregulation may contribute to early vulnerability of PLEs  Markers measured in plasma: Phosphatidylcholine, Lysophosphatidylcholine, Plasminogen b, Coagulation factor XI, Alpha1-antiplasmin  Measured variables included BMI |
| **Mico** et al. (2011) | Case-control; CAFEPS | Spain | TPD (FEP) | K-SADS-PL at admission; DSM-IV criteria at follow-up | Healthy controls | 102, 33.3% F | 95, 35.8% F | 15.61 ±0.26 | | 15.25 ±0.22 | From CAFEPS baseline data: Caucasian (85.5), Hispanic (6.4), Other (8.1) | Calculated Hedge’s g (95% CI):  Catalase –0.05 (-0.33, 0.23)  TAS -1.21 (-1.52, -0.91)  Glutathione -4.72 (-5.26, -4.17)  Lipid hydroperoxides 3.22 (2.79, 3.64)  Superoxide dismutase 0.34 (0.06, 0.62)  RBC Glutathione peroxidase 2.10 (1.76, 2.45)  Measured variables: antipsychotic medication & tobacco use did not affect results |
| Mittleman et al. (1997) | Case-control | USA | TPD (SCZ, VEOS) | DSM-III diagnosis of SCZ | OCD (n=24), ADHD (n=42) | 22, 45.45% F | 66, 16.6% F | 14.3 ±2.1 | | OCD (15.7 ±2.9) ADHD (9±1.7) | Not reported | CSF IFN-γ and IL-4 higher in SCZ than OCD and ADHD. CSF TNF in SCZ was similar to ADHD and OCD. CSF IL-2 and lymphotoxin alpha (aka TNF-β) higher in OCD than SCZ and ADHD.  Measured CSF: IL-2, IFN-γ, Lymphotoxin alpha (aka TNF-β), IL-4, IL-5, IL-10, TNF |
| Moreno et al. (2019) | Case-control; FLAMM-PEPs | Spain | TPD (SCZ) | K-SADS-PL with DSM-IV criteria | Controls could not have lifetime DSM-IV psy- chiatric disorders or a history of psychotic disorders among first-degree relatives | 27, whole sample 22.22% F | 24 | 16.85 ±1.38 | | 17.25 ±2.59 | Caucasian (92.9) | While data was available for adolescent cases and controls, the primary analyses of the source manuscript compared adolescent-onset to adult-onset psychosis and found NF-kB was higher in adolescent-onset psychosis than in adult-onset psychosis.  Markers measured included:  NF-kB, Inducible NO synthase, Cyclooxygenase 2, Prostaglandin E2, Nitrogen dioxide, Thiobarbituric acid reactive substances, IκBα, 15-Deoxy-∆-12,14-Prostaglandin J2, PPARyAct  Measured variables included: BMI, tobacco use & age of onset, cannabis use & age of onset, antipsychotic medication use |
| O'Gorman et al. (2017) | Cohort; ALSPAC | England | SP (PLE) | PLIKS interview | Age-matched individuals randomly selected from the ALSPAC cohort | 38, 78.94% F | 67, 47.79% F | Sample collected at age 11 (SD unavailable), PLIKS at age 11 (SD unavailable) | | | From ALSPAC baseline data: White (96.09) | 179 lipids identified at age 11, with 32 found to be significantly altered between control and PLE groups. Following correction, 8 remained significant- all of which were elevated in PLE group.  Markers measured include: Cholesterol ester, lysophosphatidylcholine, phosphatidylcholine, sphingomyelin, triacylglycerol, 1-monopalmitin, 2,4-Dihydroxybutanoic, D-(−)-ribofuranose, Ethanolamine, Ribitol Hydroxylamine, Sugar derivative  Matched for BMI |
| O'Gorman et al. (2017) | Cohort; ALSPAC | England | SP (PLE) | PLIKS interview | Age-matched individuals randomly selected from the ALSPAC cohort | 36, 77.77% F | 117, 50.42% F | Sample collected at age 18 (SD unavailable), PLIKS at age 18 (SD unavailable) | | | From ALSPAC baseline data: White (96.09) | At age 18, 23 lipids were significantly different between control and PLE groups, although none remained significant following correction for multiple comparisons. Findings indicate lipidome is altered in blood during childhood before development of PLEs  Markers measured include: Lysophosphatidylcholine, phosphatidylcholine, phosphoethanolamine, sphingomyelin, triacylglycerol,  1-Monopalmitin, 2-Hydroxybutyric acid, 3-Hydroxybutyric acid, Cholesterol, Citric acid, Glycine, Leucine, L-tryptophan, Oleic acid, Palmitic acid, Scyllo-inositol, Stearic acid, Sugar derivative, A144004, Sugar derivative, Threonic acid, Sugar derivative, Tocopherol α, Levoglucosan  Matched for BMI |
| **Önder** et al. (2020) | Case-control | Turkey | TPD (SCZ) | Retrospective medical record analysis of patients with SCZ | Healthy age & sex matched controls who presented for health check-up for sports license | 32, 71.87% F | 32, 75% F | 16.96 ±1.25 | | 16.84 ±1.58 | Not reported | Calculated Hedge’s g (95% CI):  NLR 0.58 (0.08, 1.07)  Neutrophil count significantly higher in patient group; however, leukocyte, haemoglobin, lymphocyte, platelet counts did not differ between groups. No significant correlation found between PANSS scores and NLR in patient group.  Markers measured in whole blood: NLR  Measured variables not included. |
| **Parellada** et al. (2012) | Case-control | Spain | TPD (FEP, SCZ) | K-SADS-PL with DSM-IV criteria | Healthy controls with similar characteristics with no history of psychiatric or neurological disorders, head trauma, or pregnancy | 34, 32% F | 34, 6% F | 15.79±1.32 | | 12.79±2.87 | Not reported | TAS did not change over time in cases or controls. Antioxidant enzymes showed no change between cases and controls. No difference in plasma GSH, TBARS, vitamin C, or vitman E levels noted. Cases had higher level of Hcy at baseline. Copper and ceruloplasmin reduced in cases. |
| **Simsek** et al. (2016a) | Case-control | Turkey | TPD (FEP) | Diagnosis of psychosis, K-SADS-PL with DSM-IV criteria | Age-matched healthy adolescents with no medical or neurological disorders | 20, 60% F | 20, 60% F | 14.5±1.6 | | 14.4±1.5 | Not reported | Calculated Hedge’s g (95% CI):  Superoxide dismutase -0.24 (-0.85, 0.37);  Serum glutathione peroxidase 0.09 (-0.52, 0.7);  CoQ -0.05 (-0.66, 0.55)  Measured variables included: BMI, smoking, height, weight, mother’s age, father’s age |
| **Simsek** et al. (2016b) | Case-control | Turkey | TPD (FEP) | DSM-IV diagnosis of acute psychosis, schizophreniform, or SCZ | Age-, gender- matched healthy controls with no medical or neurological disorders | 30, 56.6% F | 26, 53.84% F | 14.7±1.9 | | 14.5±1.9 | Not reported | Calculated Hedge’s g (95% CI): IL.17A 0.44 (-0.08, 0.97); IFN-y 0.15 (-0.37, 0.67); TNF-a 0.30 (-0.22, 0.82); IL-10 -0.53 (-1.05, 0.00); IL-6 0.19 (-0.33, 0.71); IL-4 0.07 (-0.45, 0.59); IL-2 0.51 (-0.02, 1.03)  Measured variables inlcuded: BMI, mother’s age, father’s age, number of sinlings, family history of psychiatric disorders |
| Smesny et al. (2017) | RCT; Subsequent analysis of data from Amminger 2010 | Germany | CHR-P, antipsychotic-free | UHR based on PANSS assessment | N/A | 79, 67.08% F | N/A | 16.4±2.1 | | | Caucasian (100) | Inflammatory markers were not altered in patients who transitioned to TPD within one year (n=12). IL-6 weakly inversely associated with omega-6 PUFA, highly increased in nicotine users. PUFA caused significant increase of sICAM-1.  Markers measured in plasma: sICAM, sIL-2r, IL-6  Measured variables included: BMI, nicotine use |
| **Sporn** et al. (2005) | Case-control | USA | TPD (SCZ, VEOS) | DSM-III-R /DSM-IV criteria for SCZ, onset of psychosis before age 13 | Age-, sex-, and BMI- matched controls were from a study of healthy children and adolescents | 24, 37.5% F | 21, 32.9% F | 14.3±2.2 | | 13.4±1.6 | White (71.1), African American (22.2), Hispanic (2.2), Other (4.4) | Calculated Hedge’s g (95% CI):  Plasma TNF-α 0.32 (-0.26, 0.90)  Matched for BMI |
| **Trotta** et al. (2021) | Case-control; E-Risk Longitudinal Twin Study | England/UK | SP (PLEs) | Interviews to assess 7 items related to delusions and hallucinations | E-Risk participants who reported no PLEs | 431, 50.3% F | 988, 54.2% F | Sample collected at age 18 (SD unavailable), interviews completed at age 18 (SD unavailable) | | | Not reported | Calculated Hedge’s g (95% CI):  Plasma; CRP 0.08 (-0.03, 0.19)  IL-6 -0.02 (-0.13, 0.10)  suPAR 0.13 (0.02, 0.25)  Combination of PLEs and childhood victimization (but not either alone) were associated with high CRP, IL-6, and suPAR  Measured variables include: weight, smoking status, use of inflammatory medication in 2 weeks prior to sample collection |
| **Ucuz** et al. (2020) | Case-control | Turkey | TPD (SCZ) | Medical record SCZ diagnosis, DSM-5 criteria (inpatient unit) | No psychiatric or medical illnesses determined by child psychiatrist evaluation, matched with the patient group by age and gender. | 57 (sex not reported) | 70 (sex not reported) | 15.7±1.28 | | 15.41 ±1.87 | Not reported | Calculated Hedge’s g (95% CI):  Total WBC 0.24 (-0.11, 0.59) Lymphocyte -0.21 (-0.56, 0.14) Monocyte 0.29 (-0.06, 0.63) Neutrophil 0.33 (-0.02, 0.68) Platelets 0.06 (-0.29, 0.41) MPV 0.34 (-0.01, 0.69) Neutrophil/Lymphocyte ratio 0.16 (-0.19, 0.51)  Monocyte/Lymphocyte ratio (MLR) 0.23 (-0.12, 0.58)  Platelet/Lymphocyte ratio (PLR) 0.04 (-0.31, 0.38)  Measured variables not reported |
| Wake et al. (2022) | Case-control | Japan | SP (CHR-P) | SIPS | Age- and sex-matched healthy controls | 19, 68.42% F | 21, 71.43% F | 15±2.52 | | 13±4.1 | Not reported | Calculated Hedge’s g (95% CI): Biopyrrin/Cre 0.91 (0.27, 1.55); 8OHdG/Cre -0.14 (-0.75, 0.47); Cortisol/Cre -0.33 (-0.72, 0.07); FLC/Cre -4.17 (-5.26, -3.07); Kflc/IFLC ratio –2.05 (-2.81, -1.29); Cx3CL1/Cre -0.26 (-0.87, 0.35); CRP/Cre -0.20 (-0.81, 0.41); Biopyrrin/FLC 1.11 (0.45, 1.76)  Measured variables not reported |
| **Wedervang-Resell** et al. (2020) | Cohort; Thematically Organized-Psychosis Study for Youth (Youth-TOP) | Norway | TPD (48% SCZ, 45% OPD, 6% Affective) | K-SADS-PL, PANSS | Same catchment area as the patients were randomly selected from the national population registry, no personal psychiatric history | 33, 65% F | 63, 48% F | 16.3±1.4 | | 15.9±1.4 | Not reported; “all participants were from the same catchment area with similar ethnicity” | Calculated Hedge’s g (95% CI): IL-18/IL-18BP ratio 0.83 (0.40, 1.27)  IL-18 0.81 (0.37, 1.24)  IL-18BPa -0.23 (-0.65, 0.19)  IL-18RAP -0.30 (-0.72, 0.12)  IL-18R1 -0.10 (-0.52, 0.31)  Cortisol 0.05 (-0.36, 0.47)  TC/HDL-C ratio 0.85 (0.42, 1.29)  TG 0.78 (0.35, 1.22)  CRP 0.28 (-0.14, 0.70)  Measured variables included: BMI, smoking |
| Xu et al. (2016) | Case-control | China | TPD (SCZ, FEP, antipsychotic-free | DSM-IV criteria for SCZ | Healthy community subjects without mental/neurological disorders | 14, 50% F | 13, 46.15% F | 14.4±1.1 | | 13.5±1.9 | Not reported | Calculated Hedge’s g (95% CI):  mRNA expression levels:  IL-18 0.36 (-0.38, 1.10)  IL-18R1 -1.15 (-1.95, -0.36)  IL-18RAP -0.59 (-1.34, 0.16)  Measured variables not reported |
| Zeni-Graiff et al. (2019) | Case-control; PRISMA Early Intervention Program | Brazil | CHR-P | Comprehensive Assessment of At-Risk Mental State (CAARMS) | N/A | 13, 30.8% F | N/A | 17.7±3.6 | | N/A | Caucasian (46.9) | Significant lower activity of SOD and lower GPX activity in CHR-P group compared to control group. Of note, control group had a mean age of 20.37 (SD 4.6), so case-control data was not included in the meta-analysis.  Measured markers include: Superoxide Dismutase, Glutathione Peroxidase  Measured variables not reported |
| Zhang et al. (2022) | Case-control | China | CHR-P | SIPS, psychotropically naive | Recruitment from community | 84, 41.7% F | 65, 52.3% F | 18.6±5.2 | | 18.8±5.1 | Not reported | CHR-P: IL-6 1.5 (2.7); IL-1β 0.5 (0.4)  HC: IL-6 1.5 (2.7); IL-1β 0.6 (1.2) |

***Abbreviations:***

***ARMS:*** *At-Risk Mental State*

***BMI:*** *Body mass index*

***CAARMS:*** *Comprehensive Assessment of At-Risk Mental State*

***CHR-P:*** *Clinical high-risk for psychosis*

***CRP:*** *C-reactive protein*

***CSF:*** *Cerebrospinal Fluid*

***DIS-C:*** *Diagnostic Interview Schedule for Children*

***DSM:*** *Diagnostic and Statistical Manual of Mental Disorders*

***EMR:*** *Electronic Medical Record*

***FEP:*** *First-episode psychosis*

***GAF:*** *Global Assessment of Functioning*

***GPX:*** *Glutathione Peroxidase*

***Hcy:*** *Homocysteine*

***IL:*** *Interleukin*

***IQR:*** *Interquartile range*

***K-SADS-PL:*** *Kiddie-Schedule for Affective Disorders and Schizophrenia, Present and Lifetime Version*

***MDD:*** *Major depressive disorder*

***MRNA:*** *messenger ribonucleic acid*

***NF-kB****: Nuclear factor kappa B*

***NLR:*** *Neutrophil/lymphocyte ratio*

***NOS:*** *Not otherwise specified*

***OPD:*** *Other Psychotic Disorder*

***PANSS:*** *Positive and Negative Syndrome Scale*

***PLE:*** *Psychotic-like experiences*

***PLIKS:*** *Psychosis-like symptoms*

***PUFA:*** *Polyunsaturated fatty acid*

***RBC:*** *Red blood cell*

***RCT:*** *Randomized controlled trial*

***SCID:*** *Structured Clinical Interview for DSM*

***SCZ:*** *Schizophrenia/Schizoaffective*

***SIPS:*** *Structured Interview for Psychosis-Risk Syndromes*

***SP:*** *Subthreshold Psychosis*

***SOD:*** *Superoxide Dismutase*

***SuPAR:*** *Soluble urokinase plasminogen activator receptor*

***TAS:*** *Total antioxidant status*

***TBARS:*** *Thiobarbituric acid reactive substances*

***THC:*** *Tetrahydrocannabinol*

***TNF:*** *Tumor Necrosis Factor*

***TPD:*** *Threshold Psychotic Disorder*

***UHR:*** *Ultra-high-risk*

# **Supplementary Table 2.** Studies that investigate each biomarker (immune, oxidative stress, bioactive lipids, other)

## **Supplementary Table 2a.** Immune Activation

| **IL-6** | **C-Reactive Protein (CRP)** | | | **TNF-α** | **IFN-γ** | | **IL-10** | **IL-2** | **IL-1β** | **IL-4** |
| --- | --- | --- | --- | --- | --- | --- | --- | --- | --- | --- |
| Gariup 2015 (TPD)  Föcking 2016 (TPD [FEP])  Simsek 2016b (TPD [FEP])  Berger 2018 (SP [CHR-P])  Khandaker 2014 (SP [PLE])  Smesny 2017 (CHR-P)  Lizano 2016 (FHR-P [TPD])  Chen 2021 (TPD [SCZ with FEP])  Li 2022 (TPD [SCZ, FEP])  Trotta 2021 (SP [PLE])  Zhang 2022 (CHR-P) | Wedervang-Resell 2020 (TPD)  Föcking 2016 (TPD [FEP])  Berger 2018 (SP [CHR-P])  Cullen 2017 (SP [PLE, FHR])  Khandaker 2014 (SP [PLE])  Khandaker 2021 (SP [PLE])  Chen 2021 (TPD [SCZ with FEP])  Trotta 2021 (SP [PLE])  Ceylan 2023 (TPD [SCZ]) | | | Mittleman 1997 (TPD [FEP])  Simsek 2016b (TPD [FEP])  Gariup 2015 (TPD)  Föcking 2016 (TPD [FEP])  Lizano 2016 (FHR-P [TPD])  Sporn 2005 (TPD [SCZ, VEOS])  Chen 2021 (TPD [SCZ with FEP])  Li 2022 (TPD [SCZ, FEP]) | Mittleman 1997 (TPD [FEP])  Simsek 2016b (TPD [FEP])  Gariup 2015 (TPD)  Föcking 2016 (TPD [FEP])  Lizano 2016 (FHR-P [TPD]) | | Mittleman 1997 (TPD [FEP])  Simsek 2016b (TPD [FEP])  Gariup 2015 (TPD)  Föcking 2016 (TPD [FEP])  Lizano 2016 (FHR-P [TPD]) | Mittleman 1997 (TPD [FEP])  Simsek 2016b (TPD [FEP])  Gariup 2015 (TPD)  Chen 2021 (TPD [SCZ with FEP]) | Gariup 2015 (TPD)  Föcking 2016 (TPD [FEP])  Lizano 2016 (FHR-P [TPD])  Zhang 2022 (CHR-P) | Mittleman 1997 (TPD [FEP])  Simsek 2016b (TPD [FEP])  Gariup 2015 (TPD) |
| **IL-5** | **IL-8** | | | **White Blood Cells** | **Neutrophil/Lymphocyte ratio** | | **Complement components** | **Cortisol** | **IL-12** | **IL-18** |
| Mittleman 1997 (TPD [FEP])  Gariup 2015 (TPD)  Föcking 2016 (TPD [FEP]) | Gariup 2015 (TPD)  Föcking 2016 (TPD [FEP])  Lizano 2016 (FHR-P [TPD]) | | | Ucuz 2020 (TPD [SCZ])  Falcone 2015b (TPD [FEP])  Bustan 2018 (TPD [SCZ]) | Bustan 2018 (TPD [SCZ])  Ucuz 2020 (TPD [SCZ])  Önder 2020 (TPD [EOS]) | | Föcking 2021 (SP [PLE])  English 2018 (SP [PLE]) | Wedervang-Resell 2020 (TPD)  Berger 2018 (SP [CHR-P]) | Berger 2018 (SP [CHR-P])  Lizano 2016 (FHR-P [TPD]) | Xu 2016 (TDP [SCZ, FEP])  Wedervang-Resell 2020 (TPD) |
| **IL-18R1** | **IL-18RAP** | | | **IP-10** | **Lymphocyte** | | **Lymphotoxin alpha (aka TNF-β)** | **MCP-1** | **Monocyte** | **Neutrophil** |
| Xu 2016 (TDP [SCZ, FEP])  Wedervang-Resell 2020 (TPD) | Xu 2016 (TDP [SCZ, FEP])  Wedervang-Resell 2020 (TPD) | | | Föcking 2016 (TPD [FEP])  Gariup 2015 (TPD) | Ucuz 2020 (TPD [SCZ])  Falcone 2015b (TPD [FEP]) | | Mittleman 1997 (TPD [SCZ, VEOS])  Föcking 2016 (TPD [FEP]) | Föcking 2016 (TPD [FEP])  Gariup 2015 (TPD) | Ucuz 2020 (TPD [SCZ])  Falcone 2015b (TPD [FEP]) | Ucuz 2020 (TPD [SCZ])  Falcone 2015b (TPD [FEP]) |
| **Platelets** | **VEGF** | | | **Monocyte-Lymphocyte ratio (MLR)** | **15-Deoxy-∆-12,14-Prostaglandin J2** | | **Alpha2-antiplasmin** | **Basophil** | **Coagulation factor XI** | **Cortisol/Cre** |
| Ucuz 2020 (TPD [SCZ])  Bustan 2018 (TPD [SCZ]) | Föcking 2016 (TPD [FEP])  Lizano 2016 (FHR-P [TPD]) | | | Ucuz 2020 (TPD [SCZ]) | Moreno 2019 (TPD [SCZ]) | | Madrid-Gambin 2019 (SP [PLE]) | Falcone 2015b (TPD [FEP]) | Madrid-Gambin 2019 (SP [PLE]) | Wake 2022 (SP [CHR-P]) |
| **CRP/Cre** | **CX3CL1/Cre** | | | **Cyclooxygenase 2** | **Eosinophil** | | **Eotaxin** | **Eotaxin-3** | **FLC/Cre** | **Granulocyte-macrophage colony-stimulating factor (GM-CSF)** |
| Wake 2022 (SP [CHR-P]) | Wake 2022 (SP [CHR-P]) | | | Moreno 2019 (TPD [SCZ]) | Falcone 2015b (TPD [FEP]) | | Föcking 2016 (TPD [FEP]) | Föcking 2016 (TPD [FEP]) | Wake 2022 (SP [CHR-P]) | Gariup 2015 (TPD) |
| **IκBα** | | **IFN-y-induced protein-10** | **IL-1α** | | | **IL-7** | **IL-12p40/IL23** | **IL-15** | **IL-16** | **IL-17** |
| Moreno 2019 (TPD [SCZ]) | | Gariup 2015 (TPD) | Föcking 2016 (TPD [FEP]) | | | Föcking 2016 (TPD [FEP]) | Föcking 2016 (TPD [FEP]) | Föcking 2016 (TPD [FEP]) | Föcking 2016 (TPD [FEP]) | Föcking 2016 (TPD [FEP]) |
| **IL-17A** | | **IL-18/IL-18BP ratio** | **IL-18BPa** | | | **Kflc/Cre** | **MCP-4** | **MDC** | **MIP-1α** | **MIP-1β** |
| Simsek 2016b (TPD [FEP]) | | Wedervang-Resell 2020 (TPD) | Wedervang-Resell 2020 (TPD) | | | Wake 2022 (SP [CHR-P]) | Föcking 2016 (TPD [FEP]) | Föcking 2016 (TPD [FEP]) | Föcking 2016 (TPD [FEP]) | Föcking 2016 (TPD [FEP]) |
| **Monocyte chemoattractant protein (MCP)** | | **MPV** | **NF-KappaB** | | | **Prostaglandin E2** | **sICAM** | **sICAM-1** | **sIL-2r** | **Soluble Urokinase Plasminogen Activator Receptor (suPAR)** |
| Gariup 2015 (TPD) | | Ucuz 2020 (TPD [SCZ]) | Moreno 2019 (TPD [SCZ]) | | | Moreno 2019 (TPD [SCZ]) | Smesny 2017 (CHR-P) | Föcking 2016 (TPD [FEP]) | Smesny 2017 (CHR-P) | Trotta 2021 (SP [PLE]) |
| **sVCAM-** | | **TARC** | **VEGF-C** | | | **VEGF-D** |  |  |  |  |
| Föcking 2016 (TPD [FEP]) | | Föcking 2016 (TPD [FEP]) | Föcking 2016 (TPD [FEP]) | | | Föcking 2016 (TPD [FEP]) |  |  |  |  |

## **Supplementary Table 2b.** Biomarkers of Oxidative Stress

| **Glutathione Peroxidase** | **Superoxide Dismutase** | **Glutathione** | **Total Antioxidant Status (TAS)** | **Catalase** | **Lipid Hydroperoxides** | **8OHdG/Cre** | **Biopyrrin/Cre** | **Biopyrrin/FLC** | **CoQ** | **Inducible NO synthase** |
| --- | --- | --- | --- | --- | --- | --- | --- | --- | --- | --- |
| Mico 2011 (TPD [FEP])  Simsek 2016a (TPD [FEP])  Zeni-Graiff 2019 (CHR-P)  González-Pinto 2012 (FHR-P)  Parellada 2012 (TPD [SCZ, FEP]) | Mico 2011 (TPD [FEP])  Simsek 2016a (TPD [FEP])  Zeni-Graiff 2019 (CHR-P)  González-Pinto 2012 (FHR-P)  Parellada 2012 (TPD [SCZ, FEP]) | Mico 2011 (TPD [FEP])  Martínez-Cengotitabengoa 2014 (FEP)  González-Pinto 2012 (FHR-P)  Parellada 2012 (TPD [SCZ, FEP]) | Mico 2011 (TPD [FEP])  González-Pinto 2012 (FHR-P)  Garcia 2018 (TPD [FEP])  Parellada 2012 (TPD [SCZ, FEP]) | Mico 2011 (TPD [FEP])  González-Pinto 2012 (FHR-P)  Parellada 2012 (TPD [SCZ, FEP]) | Mico 2011 (TPD [FEP])  González-Pinto 2012 (FHR-P) | Wake 2022 (SP [CHR-P]) | Wake 2022 (SP [CHR-P]) | Wake 2022 (SP [CHR-P]) | Simsek 2016a (TPD [FEP]) | Moreno 2019 (TPD [SCZ]) |
| **Thioredoxin-1** | **Homocysteine** | **Vitamin C, E** |  |  |  |  |  |  |  |  |
| Lundberg 2022 (SP [PLE]) | Parellada 2012 (TPD [SCZ, FEP]) | Parellada 2012 (TPD [SCZ, FEP]) |  |  |  |  |  |  |  |  |

## **Supplementary Table 2c.** Bioactive Lipids including Fatty Acids and Phospholipids

| **Lysophosphatidylcholine (LPC)** | **Phosphatidylcholine** | **1-Monopalmitin** | **2-Hydroxybutyric acid** | **2,4-Dihydroxybutanoic acid** | **A-Linolenic acid** | **Arachidonic acid** | **Cholesterol ester (CE)** |
| --- | --- | --- | --- | --- | --- | --- | --- |
| O’Gorman 2017 (SP [PLE])  Madrid-Gambin 2019 (SP [PLE]) | O’Gorman 2017 (SP [PLE])  Madrid-Gambin 2019 (SP [PLE]) | O’Gorman 2017 (SP [PLE]) | O’Gorman 2017 (SP [PLE]) | O’Gorman 2017 (SP [PLE]) | Amminger 2010 (SP [CHR-P]) | Amminger 2010 (SP [CHR-P]) | O’Gorman 2017 (SP [PLE]) |
| **D-(−)-ribofuranose** | **Docosapentaenoic acid** | **Docoshexaenoic acid** | **Eicosapentaenoic acid** | **Erythrocyte membrane fatty acids** | **Ethanolamine** | **Hydroxylamine** | **Linoleic acid** |
| O’Gorman 2017 (SP [PLE]) | Amminger 2010 (SP [CHR-P]) | Amminger 2010 (SP [CHR-P]) | Amminger 2010 (SP [CHR-P]) | Berger 2016 (SP [CHR-P]) | O’Gorman 2017 (SP [PLE]) | O’Gorman 2017 (SP [PLE]) | Amminger 2010 (SP [CHR-P]) |
| **Oleic acid** | **Ribitol** | **Sphingomyelin** | **TC/HDL-C ratio** | **Thiobarbituric acid reactive substances** | **Tie-2** | **Triacylglycerol** | **Triglycerides (TG)** |
| O’Gorman 2017 (SP [PLE]) | O’Gorman 2017 (SP [PLE]) | O’Gorman 2017 (SP [PLE]) | Wedervang-Resell 2020 (TPD) | Moreno 2019 (TPD [SCZ]) | Föcking 2016 (TPD [FEP]) | O’Gorman 2017 (SP [PLE]) | Wedervang-Resell 2020 (TPD) |

## **SupplementaryTable 2d.** Other Biomarkers

| **Basic Fibroblast Growth Factor (bFGF)** | **PIGF** | **sFlt-1** | **Brain Derived Neurotrophic Factor (BDNF)** | **Niacin sensitivity** | **Nitrogen dioxide** | **Platelet-Lymphocyte ratio (PLR)** | **PPARy** |
| --- | --- | --- | --- | --- | --- | --- | --- |
| Föcking 2016 (TPD [FEP])  Lizano 2016 (FHR-P [TPD]) | Föcking 2016 (TPD [FEP])  Lizano 2016 (FHR-P [TPD]) | Föcking 2016 (TPD [FEP])  Lizano 2016 (FHR-P [TPD]) | Lizano 2016 (FHR-P [TPD]) | Berger 2016 (SP [CHR-P]) | Moreno 2019 (TPD [SCZ]) | Ucuz 2020 (TPD [SCZ]) | Moreno 2019 (TPD [SCZ]) |
| **S100B** | **SAA** |  |  |  |  |  |  |
| Falcone 2015a (TPD [FEP]) | Föcking 2016 (TPD [FEP]) |  |  |  |  |  |  |

# **Supplementary Table 3.** Biosample collection, storage, and analysis descriptions

| **Study** | **Cohort (if applicable)** | **Sample Type & Biomarkers** | **Collection** **Procedure Description** | **Storage & Analysis Procedure Description** |
| --- | --- | --- | --- | --- |
| Amminger et al. (2010) |  | Whole blood: RBC membrane A-Linolenic acid (parent fatty acid (FA) to omega-3 FA), Eicosapentaenoic acid, Docosapentaenoic acid, Docoshexaenoic acid, Linoleic acid, Arachidonic acid | Time of collection not reported, fasting sample | N/A |
| Berger et al. (2016) |  | Whole blood, plasma: Niacin sensitivity | N/A | N/A |
| Berger et al. (2018) | NEURAPRO | Plasma: CRP, IL-6, IL-12  Saliva: cortisol | Saliva collected at awakening, fasting blood sample | Samples stored at -80°C until analysis |
| **Bustan** et al. (2018) |  | Whole blood: Total WBC, platelets, neutrophil/lymphocyte ratio | *Electronic medical record review of clinical labs* | *Electronic medical record review of clinical labs* |
| Ceylan et al. (2023) |  | Serum: CRP | 10mL Blood samples collected at 9am from antecubital vein after 12 hour overnight fasting period. Centrifuged at 2000xg for 15 min after incubation period of 30min. | Serum samples stored at –20 degrees Celsius and then analyzed with Human NEFL ELISA kit |
| **Chen** et al. (2021) |  | Serum:  IL-6, TNF- α, CRP, IL-2 | Fasting serum samples collected in serum separator tubes, clotted for 30 minutes | Samples stored at -80°C until assay |
| **Cullen** et al. (2017) |  | Saliva: CRP | Saliva collected at awakening, 15min, 30min, and 60min after awakening, at 12:00 and 20:00; 30min fasting prior to each sample collection. | Samples stored in participants’ home freezers until collection, thawed for centrifugation, then stored at -20°C at laboratory (storage up to 5 years; mean lapse of time = 1186 days) |
| English et al (2018) | ALSPAC | Plasma: Protein expression- Alpha-2-macroglobulin, Tenascin-X, Ig mu chain C region, Tetranectin, Cholinesterase, Gelsolin, Extracellular matrix protein 1, Insulin-like growth factor II, C4b-binding protein beta chain, Di-N-acetylchitobiase, Thyroxine-binding globulin, Vascular cell adhesion protein 1, Lumican, Phosphatidylinositol-glycan- specific phospholipase D, Hyaluronan-binding protein 2, Ig gamma-3 chain C region, Selenoprotein P, Complement factor H, Pigment epithelium-derived factor, Coagulation factor V, Coagulation factor IX, Insulin-like growth factor- binding protein 3, Coagulation factor XII, Complement factor D, Complement factor I, Lysosome-associated membrane glycoprotein 2, Cartilage acidic protein 1,  Procollagen C-endopeptidase enhancer 1, Afamin, Plasminogen, Collagen alpha-3(VI) chain, Complement component C7, Apolipoprotein A-11, CD109 antigen, actin, cytoplasmic 2, monocyte differentiation antigen CD14, Plasma kallikrein, kininogen-1, coagulation factor V, alpha-2 antiplasmin, complement C1s subcomponent, prothrombin, apolipoprotein A-IV, Ig kappa chain V-II region TEW, lymphatic vessel endothelial hyaluronic acid receptor 1, coagulation factor XIII A chain, Beta-2-glycoprotein 1, complement component C6, complement C1r subcomponent, clusterin, cartilage oligomeric matrix protein, carboxypeptidase N subunit 2, adiponectin, attractin, vasorin, retinoic acid receptor responder protein 2 | Nonfasting blood collected at 11 years of age | Samples stored on ice for a maximum of 90 min until processed; Plasma stored in aliquots at -80°C; All samples underwent a single freeze-thaw cycle to allow aliquotting prior to the study |
| Falcone et al. (2015a) |  | Serum: S100B | N/A | Serum stored at -80°C until entire sample was achieved |
| **Falcone** et al. (2015b) |  | Whole blood: Total WBC, basophil, eosinophil, lymphocyte, monocyte, neutrophil    Serum: S100B, IL-1α, IL-1β, IL-2, IL-4, IL-6, IL-8, IL-10, IFN-γ, TNF-a, C-Reactive Protein (CRP), TNF-β, and IL-5 | Blood collected between 9am-11am | Serum separated and stored at −80°C until assayed |
| Föcking et al. (2016) |  | Plasma: CRP, Eotaxin, Eotaxin-3, IFN-γ, IL-10, IL-12p40/IL23, IL-15, IL-16, IL-17, IL-1α, IL-1β, IL-5, IL-6, IL-7, IL-8, IP-10, MCP-1, MCP-4, MDC, MIP-1α, MIP-1β, PIGF, SAA, TARC, TNF-α, Lymphotoxin alpha (aka TNF-β), Tie-2, VEGF, VEGF-C, VEGF-D, bFGF, sFlt-1, sICAM-1, sVCAM-1 | N/A | N/A |
| Föcking et al. (2021) | ALSPAC | Plasma: Vitronectin, Complement C1r, Subcomponent-like protein, Complement componenet C8 beta chain, complement component C8 alpha chain, Complement factor H, Complement C5, C4b-binding protein alpha chain, Complement C2, Mannan-binding lectin serine protease 1, Complement C1s subcomponent, Complement factor B, Complement component C8 gamma chain, Complement C1q subcomponent subunit A, Complement C4-A, Complement C1q subcomponenet subunit B, Complement factor 1, Clusterin, Complement C1r subcomponent, Compliment component C6, Compement C1q subcomponent subunit C, C4b-binding protein beta chain, Complement factor H-related protein 5, Complement component C9, Complement factor D, Plasma protease, C1 inhibitor, Mannose-binding protein C, Complement C4-B, Complement component C7, Complement C3 | Non-fasting blood samples collected at ~12 years of age | Samples stored on ice for a maximum of 90 min until processed; plasma stored in aliquots at -80°C; one additional freeze-thaw cycle to allow aliquoting. |
| **Gariup** et al. (2015) |  | Whole blood: WBC and differential measured    Serum: CRP, GM-CSF, IFN-γ, IL-1b, IL-2, IL-4, IL-5, IL-6, IL-8, IL-10, TNF-α, IP-10, MCP-1  *Only IL-6 data had median and interquartile ranges that could be used for meta-analysis | Fasting blood samples collected early morning | Serum stored at -80°C until time of assay (between 12-30 months) |
| **Gonzalez-Pinto** et al. (2012) |  | Plasma: TAS, lipid hydroperoxides  RBC: Glutathione, catalase, superoxide dismutase, glutathioine peroxidase | N/A | RBC, plasma were separated by centrifugation and immediately frozen at -80°C until analysis |
| **Khandaker** et al. (2014a) | ALSPAC | Serum: IL-6, CRP | Non-fasting blood samples collected | Samples were immediately spun and frozen at −80°C. Median of 7.5 years in storage with no previous freeze-thaw cycles during this period. |
| Khandaker et al. (2021) | ALSPAC | Serum: CRP | Fasting blood samples collected morning (overnight fast) or afternoon (6 hour fast) | Samples were immediately spun, frozen and stored at −80 °C, which were analysed within 3–9 months of blood sampling with no freeze-thaw cycles in between. |
| **Li** et al. (2022) |  | Plasma: IL-6, TNF- α | Blood samples collected between 20:00-22:00 | Samples stored at −80 °C until assay |
| Lizano et al. (2016) |  | Plasma: IFN-γ, IL-1β**,** IL-6, IL-8  IL-10, IL-12, TNF-α, PIGF, VEGF | Fasting samples collected at baseline visit | Plasma aliquots frozen at −80°C until use in immunoassays to avoid freeze/thaw cycles. |
| Lundberg et al. (2022) |  | Plasma: Trx1, latent TGF-b1, IgG, CD62P/P-selectin and Hb | Blood samples drawn from antecubital vein between 9:00am-10:00am after overnight fasting. Samples centrifuged within 60 minutes of drawing for 20 minutes at 1500xg at 4 degrees Celsius to remove all platelets | Plasma phase collected and stored at –70 degrees Celsius until use. An ELISA system was generated by replacing anti-Trx1 antibodies with murine mAbs with non-matching specificty. The replacing antibodieswere matched with regard to the subclass of the anti-Trx1 mAbs. To assess the potential problem with HA, a comparison was made with plasma samples diluted in incubation buffer. |
| Madrid-Gambin et al. (2019) | ALSPAC | Plasma: Phosphatidylcholine, Lysophosphatidylcholine,  Plasminogen b, Coagulation factor XI, Alpha2-antiplasmin | Non-fasting blood samples collected | Samples were stored on ice for a maximum of 90 minutes until processed. Postcentrifugation, the samples were stored at -80C until further analyses. |
| Martinez-Cengotitabengoa et al. (2014) | CAFEPS | Plasma TAS | Blood samples collected at time of admission to inpatient psychiatric unit | Samples stored at −80°C until analysis |
| **Mico** et al. (2011) | CAFEPS | Plasma: TAS, Lipid peroxidation assay for lipid hydroperoxides    RBC: Glutathione, Glutathione peroxidase, Superoxide dismutase, Catalase | Non-fasting blood samples collected immediately following enrollment | Samples stored at -80°C until analysis |
| Mittleman et al. (1997) |  | Measured CSF: IL-2, IFN-γ, Lymphotoxin alpha (aka TNF-β), IL-4, IL-5, IL-10, TNF | Spinal fluid samples obtained via lumbar puncture between 7:30 and 9:30 a.m following >8 h of bed rest | Samples stored at -70°C until assay |
| Moreno et al. (2019) | FLAMM-PEPs | Plasma: Prostaglandin E2, 15-Deoxy-∆-12,14-Prostaglandin J2, Nitrogen dioxide, Thiobarbituric acid reactive substances    Peripheral blood mononuclear cells:  NF-KappaB, Inducible NO synthase,  Cyclooxygenase 2, PPARyAct, IκBα | Fasting blood sample collected between 8:00-10:00am | Samples stored at –80ºC |
| O'Gorman et al. (2017) | ALSPAC | Plasma: CE(18:2), CE(18:2)+unknown, LPC(16:1), LPC(18:1), LPC(18:2), LPC(20:3), LPC(20:4), PC(16:0e/18:1(9Z)), PC(30:0), PC(32:0), PC(32:1), PC(32:2), PC(34:2), PC(36:2), PC(36:3), PC(36:4, PC(38:2), PC(38:4), PC(40:6), PC(O-32:0), PC(O-32:1), PC(O-34:2), PC(O-34:3), PC(O-36:2), PC(O-36:3), PC(O-38:6), SM(d18:0/16:0), SM(d18:1/24:0), SM(d41:1), SM(d42:2), TG(56:7), 1-monopalmitin, 2,4-Dihydroxybutanoic, D-(−)-ribofuranose, Ethanolamine, Ribitol Hydroxylamine, Sugar derivative | Non-fasting blood samples collected at age ~11 | Samples stored on ice for a maximum of 90 min until processed. Post centrifugation, the plasma samples were stored at −80 °C until further analyses. |
| O'Gorman et al. (2017) | ALSPAC | Plasma: LPC(16:0), LPC(16:1), LPC(22:6), PC(31:0), PC(32:1), PC(33:1), PC(35:1), PC(36:2), PC(36:4), PC(37:4), PC(38:6), PC(40:6), PC(40:7), PC(O-38:4), PC(O-40:6), PC(O-42:3), PE(P-18:0/22:6), SM(d18:0/16:0), SM(d32:1), SM(d33:1), TG(49:3), TG(51:2), TG(56:7),   1-Monopalmitin, 2-Hydroxybutyric acid, 3-Hydroxybutyric acid, Cholesterol, Citric acid, Glycine, Leucine, L-tryptophan, Oleic acid, Palmitic acid, Scyllo-inositol, Stearic acid, Sugar derivative, A144004, Sugar derivative, Threonic acid, Sugar derivative, Tocopherol α, | Fasting blood samples collected at age ~18 | Samples stored on ice for a maximum of 90 min until processed. Post centrifugation, the plasma samples were stored at −80 °C until further analyses. |
| **Önder** et al. (2020) |  | Whole blood: Neutrophil/lymphocyte ratio | *Electronic medical record review of clinical labs* | *Electronic medical record review of clinical labs* |
| **Parellada et al. (2012)** |  | Plasma: TAS, glutathione, homocysteine, catalase, superoxide dismutase, glutathione peroxidase, antioxidants, lipid peroxidation | Fasting 9mL blood samples collected | Samples underwent immediate centrifugation, plasma & whole blood aliquots stored frozen at −80 °C |
| **Simsek** et al. (2016a) |  | Serum: Superoxide dismutase, Glutathione peroxidase, CoQ | Blood samples collected between 9:00am-12:00pm | Samples were allowed to clot for 15 min, then centrifuged at 5,000 rpm for 6 min. Samples stored at −80 °C until analysis. |
| **Simsek et al.** (2016b) |  | Serum: IL.17A, IFN-y, TNF-α, IL-10, IL-6, IL-4, IL-2 | Nonfasting blood samples collected between 10:00am-12:00pm | Samples stored at room temperature for 30 minutes for coagulation. Blood samples then centrifuged at 1000g for 10 minutes. Samples stored at −80 °C until analysis. |
| Smesny et al. (2017) |  | Plasma: sICAM, sIL-2r, IL-6 | Fasting blood samples collected | Samples stored at −72 °C until analysis; no additional freeze-thaw cycles |
| **Sporn** et al. (2005) |  | Plasma: TNF-α | Fasting blood samples collected between 6:00am-7:00am | Samples stored at −80 °F until analysis (storage temperature reported in degrees Fahrenheit in published manuscript) |
| **Trotta** et al. (2021) | E-Risk | Plasma: Su-PAR C-reactive protein, IL-6 | N/A | Samples stored at −80 °C until analysis |
| **Ucuz** et al. (2020) |  | Total WBC, Lymphocyte, Monocyte, Neutrophil, Platelets, MPV, Neutrophil/Lymphocyte ratio, Monocyte-Lymphocyte ratio, Platelet-Lymphocyte ratio | *Electronic medical record review of clinical labs* | *Electronic medical record review of clinical labs* |
| Wake et al. (2022) |  | Urine: Biopyrrin/Cre, 8oHdG/Cre, Cortisol/Cre, FLC/Cre, Kflc/IFLC ratio, Cx3Cl1/Cre, CRP/Cre, Biopyrrin/FLC | Spot urine samples collected | Samples stored at −80 °C until analysis |
| **Wedervang-Resell** et al. (2020) | Youth-TOP | Plasma: IL-18/IL-18BP ratio  IL-18, IL-18BPa, IL-18RAP, IL-18R1    Serum: Cortisol, TC/HDL-C ratio, TG, C-reactive protein | Fasting blood samples collected between 8:10am-11:20am | For hormone and biochemical analyses, serum  was separated within two hours. For immunoassays, blood was taken using EDTA vials and the plasma was isolated the next working day. Samples stored at −80 °C until analysis. |
| Xu et al. (2016) |  | mRNA expression levels: IL-18, IL-18R1, IL-18RAP | N/A | N/A |
| Zeni-Graiff et al. (2019) | PRISMA Early Intervention Program | Serum: Superoxide Dismutase  Glutathione Peroxidase | Fasting blood samples collected between 8:00am-10:00am | After collection, blood samples were immediately placed on ice, allowed to clot for at least 30 min at room temperature, and centrifuged at 3200 rpm for 10 min.  Samples stored at −80 °C until analysis (maximum 2 years) |
| Zhang et al. (2022) |  | Serum: IL-6, IL-1β | Fasting blood samples drawn in morning, kept at room temperature for 1 hour, and centrifuged (1710xg, 20min, 4 degrees Celsus) for serum separation. Serum then separated and stored at –80 degrees Celsius until analysis | Serum separated and stored at –80 degrees Celsius until analysis |

**Bolded** studies were included in the meta-analyses

***Abbreviations:***

***ALSPAC:*** *Avon Longitudinal Study of Parents and Children*

***CAFEPS:*** *Child and Adolescent First-Episode Psychosis Study*

***CRP:*** *C-reactive protein*

***E-Risk:*** *Environmental risk longitudinal twins study*

***FLAMM-PEPs:****“Inflammatory alterations in schizophrenia: the search for biological markers in first-episode psychosis” study*

***HDL-C:*** *high-density cholesterol*

***IL:*** *interleukin*

***NEURAPRO:*** *A multi-centre RCT of omega-3 polyunsaturated fatty acids versus placebo in young people at ultra-high risk of psychotic disorders*

***RBC:*** *red blood cell (count)*

***SD:*** *standard deviation*

***TAS:*** *Total antioxidant status*

***TC:*** *Total cholesterol*

***TG:*** *triglycerides*

***TNF:*** *tumor necrosis factor*

***VEGF:*** *vascular endothelial growth factor*

***WBC:*** *white blood cell (count)*

***Youth-TOP:*** *Thematically-organized psychosis study for youth*

# **Supplementary Figure 2.** Funnel plots for threshold psychosis

Nonparametric trim-and-fill analysis of publication bias revealed no changes in estimated effect size

Nonparametric trim-and-fill analysis of publication bias revealed no changes in estimated effect size

Nonparametric trim-and-fill analysis of publication bias revealed no changes in estimated effect size

Nonparametric trim-and-fill analysis of publication bias revealed no changes in estimated effect size

Nonparametric trim-and-fill analysis of publication bias revealed no changes in estimated effect size

Nonparametric trim-and-fill analysis of publication bias revealed no changes in estimated effect size

Nonparametric trim-and-fill analysis of publication bias revealed no changes in estimated effect size

# **Supplementary Table 4.** Quality assessment scores by study

| The Newcastle-Ottawa Scale (NOS) for assessing the quality of studies in meta-analyses | | | | | | | | | |
| --- | --- | --- | --- | --- | --- | --- | --- | --- | --- |
| **Study** | **Selection** | | | | **Comparability** | **Exposure/Outcome** | | | **Total Quality score** |
| Berger et al. (2016) | 1 | 1 | 0 | 1 | 2 | 0 | 1 | 1 | 7 |
| Berger et al. (2018) | 1 | 1 | 1 | 1 | 2 | 1 | 1 | 0 | 8 |
| Bustan et al. (2018) | 1 | 1 | 1 | 1 | 1 | 0 | 1 | 1 | 7 |
| Ceylan et al. (2023) | 1 | 1 | 1 | 0 | 0 | 1 | 1 | 1 | 6 |
| Chen et al. (2021) | 1 | 0 | 0 | 1 | 2 | 1 | 1 | 1 | 7 |
| Cullen et al. (2017) | 1 | 1 | 1 | 1 | 2 | 1 | 1 | 1 | 9 |
| English et al (2018) | 1 | 1 | 1 | 1 | 2 | 1 | 1 | 1 | 9 |
| Falcone et al. (2015a) | 1 | 0 | 0 | 1 | 2 | 1 | 1 | 1 | 7 |
| Falcone et al. (2015b) | 1 | 0 | 1 | 1 | 2 | 1 | 1 | 0 | 7 |
| Föcking et al. (2016) | 1 | 0 | 0 | 1 | 2 | 0 | 1 | 0 | 5 |
| Föcking et al. (2021) | 1 | 1 | 1 | 1 | 1 | 1 | 1 | 1 | 8 |
| Garcia et al. (2018) | 1 | 1 | 1 | 1 | 2 | 1 | 1 | 1 | 9 |
| Gariup et al. (2015) | 1 | 1 | 1 | 1 | 2 | 1 | 1 | 1 | 9 |
| Gonzalez-Pinto et al. (2012) | 1 | 1 | 1 | 1 | 1 | 1 | 1 | 0 | 7 |
| Khandaker et al. (2014) | 1 | 1 | 1 | 1 | 2 | 1 | 1 | 1 | 9 |
| Khandaker et al. (2021) | 0 | 1 | 1 | 0 | 2 | 1 | 1 | 0 | 6 |
| Li et al. (2022) | 1 | 1 | 1 | 1 | 0 | 1 | 1 | 0 | 6 |
| Lizano et al. (2016) | 1 | 1 | 1 | 1 | 1 | 1 | 1 | 1 | 8 |
| Lundberg et al. (2022) | 1 | 0 | 1 | 1 | 0 | 1 | 1 | 1 | 6 |
| Madrid-Gambin et al. (2019) | 1 | 1 | 1 | 1 | 2 | 1 | 1 | 1 | 9 |
| Mico et al. (2011) | 1 | 1 | 1 | 1 | 1 | 1 | 1 | 1 | 8 |
| Mittleman et al. (1997) | 1 | 1 | 0 | 0 | 0 | 1 | 1 | 1 | 5 |
| Moreno et al. (2019) | 1 | 1 | 1 | 1 | 2 | 1 | 1 | 1 | 9 |
| O'Gorman et al. (2017) | 1 | 1 | 1 | 1 | 1 | 1 | 1 | 1 | 8 |
| Önder et al. (2020) | 1 | 0 | 0 | 0 | 2 | 0 | 0 | 1 | 4 |
| Parellada et al. (2012) | 1 | 1 | 1 | 1 | 2 | 1 | 1 | 1 | 9 |
| Simsek et al. (2016a) | 1 | 1 | 0 | 0 | 2 | 1 | 1 | 1 | 7 |
| Simsek et al. (2016b) | 1 | 1 | 0 | 1 | 0 | 1 | 1 | 1 | 6 |
| Smesny et al. (2017) | 1 | 1 | 1 | 1 | 2 | 1 | 1 | 1 | 9 |
| Sporn et al. (2005) | 1 | 1 | 1 | 1 | 2 | 1 | 0 | 1 | 8 |
| Trotta et al. (2021) | 1 | 1 | 1 | 1 | 2 | 1 | 1 | 1 | 9 |
| Ucuz et al. (2020) | 0 | 1 | 1 | 1 | 2 | 0 | 0 | 1 | 6 |
| Wake et al. (2022) | 1 | 0 | 0 | 0 | 2 | 0 | 0 | 1 | 4 |
| Wedervang-Resell et al. (2020) | 1 | 1 | 1 | 1 | 2 | 1 | 1 | 1 | 9 |
| Xu et al. (2016) | 1 | 0 | 1 | 1 | 2 | 0 | 1 | 1 | 7 |
| Zeni-Graiff et al. (2019) | 1 | 1 | 1 | 1 | 2 | 1 | 1 | 1 | 9 |
| Zhang et al. (2022) | 1 | 1 | 1 | 0 | 1 | 1 | 1 | 1 | 7 |

**RoB 2 tool (revised tool for Risk of Bias in randomized trials)**

|  | Randomisation | Assignment of intervention | Adherence to intervention | Missing outcome data | Outcome measurement | Selection of reported outcome | Overall risk |
| --- | --- | --- | --- | --- | --- | --- | --- |
| Amminger et al. (2010) | Low | Low | Low | Low | Low | Low | Low |
